# Does High-Intensity Interval Training Increase Muscle Strength, Muscle Mass, and Muscle Endurance? A Systematic Review and Meta-Analysis

**DOI:** 10.3390/sports13090293

**Published:** 2025-09-01

**Authors:** Lucas Wiens, Justin M. Losciale, Matthew D. Fliss, Max J. Abercrombie, Darius Darabi, Jedd Li, Rowan Barclay, Cameron J. Mitchell

**Affiliations:** 1 School of Kinesiology, Faculty of Education, The University of British Columbia, 2135 Wesbrook Mall Vancouver, Vancouver, BC V6T 1Z3, Canada; wiensl55@student.ubc.ca (L.W.); matthew.fliss@ubc.ca (M.D.F.); max.abercrombie@ubc.ca (M.J.A.); darabidarius@gmail.com (D.D.); jeddli2002@gmail.com (J.L.); rowanbarclay@gmail.com (R.B.); 2 Informatics, Decision-Enhancement, and Analytic Sciences Center of Innovation, George E. Wahlen Department of Veterans Affairs Medical Center, 500 Foothill Dr, Salt Lake City, UT 84148, USA; 3 Division of Epidemiology, Department of Internal Medicine, University of Utah School of Medicine, Salt Lake City, UT 84132, USA

**Keywords:** high-intensity interval training, sprint interval training, muscle strength, muscle hypertrophy, muscle endurance

## Abstract

High-intensity/sprint interval training (HIIT/SIT) improves aerobic and anaerobic performance, but it is unknown if HIIT/SIT increases strength, muscle mass/size, and muscle endurance (ME). We aimed to determine if HIIT/SIT increases strength, muscle mass/size, and ME. Databases (Ovid Medline, Sport Discus, EMBASE, and CINAHL) and the gray literature (Google Scholar) were searched for original research articles investigating the impact of HIIT/SIT on strength, muscle mass/size, and ME (23 March 2025). The risk of bias (ROB) was assessed via the Cochrane ROB 2 Tool. Meta-analyses were performed when three or more randomized controlled trials compared HIIT/SIT to a common comparator. Fifty-four studies were included (*N* = 1136). Twenty-five studies had a high ROB, while twenty-nine had some concerns. Standardized mean differences (SMD) (95% CI) of 0.16; (−0.09, 0.40), 0.33; (−0.21, 0.87) were observed for meta-analyses comparing the effect of HIIT/SIT to moderate intensity continuous training (MICT) and non-exercise controls (CON) on FFM, respectively. A meta-analysis comparing the effect of HIIT/SIT to resistance training (RT) on leg press strength yielded a SMD of −0.82; 95% CI: (−1.97, 0.33). HIIT/SIT may induce slightly greater gains than MICT and CON for FFM, while RT is likely superior to HIIT/SIT for improving leg press strength. However, the certainty of evidence is low, and 95% CIs intersect zero for all analyses.

## 1. Introduction

The American College of Sports Medicine recommends 150 min of moderate-intensity aerobic activity and two sessions of muscle strengthening exercises per week [1]. ~50.5% of individuals meet aerobic exercise guidelines; however, only ~30% of individuals meet muscle strengthening guidelines, and just ~20% meet both aerobic and muscle strengthening guidelines [2]. The most cited barrier to exercise is “lack of time” [3], but if benefits associated with both resistance training (RT) and aerobic training could be achieved more efficiently, this barrier could be lifted.

A time efficient exercise method is high-intensity interval training (HIIT). HIIT intervals last 1–4 min and typically range from 80 to 95% of maximal effort, with constant power output maintained [4]. Sprint interval training (SIT) is performed at maximal effort with intervals lasting ~10–30 s, using fixed loads, with performance usually declining during subsequent efforts [4]. HIIT/SIT induces similar or greater aerobic fitness improvements relative to MICT in less time [4], improves anaerobic performance [5,6], and results in comparable levels of fatigue and metabolic stress as RT [7,8]. Therefore, HIIT/SIT may be time efficient methods to improve both muscular and aerobic fitness. It is currently unknown if HIIT/SIT promotes increases in strength, muscle mass/size, or ME.

Aerobic training is not typically considered to be an anabolic stimulus; however, some reports of hypertrophy in response to aerobic training exist given sufficient volume and intensity [9]. The two most important training variables for inducing muscle hypertrophy are training volume (reps × sets × load) and training in proximity to momentary failure (high levels of perceived effort) [10,11,12]. HIIT/SIT induce high levels of perceived effort [13], while SIT results in performance declines, indicating fatigue (task failure) [14]. Typical HIIT/SIT also demands a greater training volume relative to most RT interventions [7,8]. Therefore, if high enough intensities are achieved during HIIT/SIT, hypertrophy may result.

Despite the high-intensity nature of HIIT/SIT, minimal work has examined the impact of HIIT/SIT interventions on muscle hypertrophy, with most studies assessing whole-body changes in fat-free mass (FFM). A narrative review on this topic [15] detailing the acute and chronic impact of HIIT/SIT on muscle hypertrophy at the molecular, cellular, muscular, and whole-body level demonstrated that HIIT/SIT may activate similar hypertrophic signaling pathways as RT [16,17] and elevate both myofibrillar and sarcoplasmic protein synthesis following acute exercise bouts [18]. The authors concluded that HIIT/SIT elicits changes at the transcriptional and translational level associated with muscle hypertrophy but did not systematically review HIIT/SIT-induced changes in muscle size. The only meta-analysis examining this topic [19] compared 4 weeks or more of low-volume HIIT/SIT to MICT and found no difference in FFM, but no comparisons were made to control groups, and assessments of localized muscle hypertrophy were not included.

Improvements in muscle strength are regulated by neural adaptions and increased muscle physiological cross-sectional area, induced via mechanical tension, typically achieved via RT [20,21,22]. In practice, lifting heavier loads (a higher percentage of an individual’s one-repetition maximum (1-RM)) yields greater gains in muscle strength compared to lifting lighter loads for more repetitions [23]. However, strength gains are still observed following lower load RT [24,25,26]. Peak pedal forces during HIIT/SIT cycling are estimated to be ~200–500 N/leg [27], while peak isometric leg press force has been documented to be ~2000–2500 N/leg in similar populations [28]. Therefore, peak forces during HIIT/SIT likely range from ~10–25% of MVC/1-RM. Previous work observed strength gains from RT at ~20% 1-RM [10], suggesting that loads encountered during HIIT/SIT cycling may be sufficient to induce strength gains.

Muscle endurance (ME) is the ability of a given muscle or muscle group to resist fatigue when performing resistance exercise at submaximal loads and is vital for many activities of daily life and physical performance [29]. It can be assessed at any load but is typically tested at loads corresponding to 30–60% 1-RM and can be measured in absolute or in relative terms [29]. Absolute ME reflects training-induced changes in work capacity, while relative ME is a measure of performance fatigability [29]. Low-load RT improves both absolute and relative ME when tested at low relative loads [24]. Muscle strength, mitochondrial function and content, muscle capillarization, and habituation to exercise-induced discomfort are theorized regulatory factors of ME performance [29]. HIIT/SIT increases mitochondrial function, mitochondrial content, muscle capillarization and induces high levels of perceived exertion [4]. Therefore, HIIT/SIT may improve ME; however, minimal work has investigated this.

Due to the barrier of time, a majority of individuals do not meet exercise guidelines, especially muscle strengthening guidelines [3,30,31]. The benefits of HIIT/SIT on aerobic fitness are unequivocal and are more time efficient than MICT [4]. However, it is unknown if HIIT/SIT can increase muscle mass/size, strength, or ME. The aim of this systematic review and meta-analysis was to determine if HIIT/SIT improves muscle strength, muscle mass/size, and muscle endurance in healthy adults.

## 2. Methods

### 2.1. Protocol and Registration

This study is a systematic review and meta-analysis. The study protocol was prospectively registered on PROSPERO—(Can High-Intensity Interval Training Induce Gains in Muscle Strength, Muscle Hypertrophy, and Local Muscle Endurance)—CRD42023400067—15 February 2023, and is reported following the PRISMA guidelines [32,33] (Figure S1).

### 2.2. Eligibility Criteria

Studies were included if they measured the effect of a HIIT/SIT intervention(s) compared to either a non-exercise or other exercise control group(s) on either muscle strength, muscle size/mass, or ME in healthy individuals (including obese individuals), aged 18–80, and of any sex or training status. HIIT/SIT interventions were required to span a minimum duration of 6 weeks, with no fewer than 12 training sessions completed. Only aerobic training interventions (i.e., cycling, running, rowing) with intervals lasting 10 s to 4 min at an intensity ≥ 80% maximum heart rate (HR)/VO_2_peak/work-peak were included in this review. Only randomized control trials (RCTs) were included in the meta-analysis. No restrictions were be placed on the types of studies included in the systematic review. Only peer-reviewed studies of English language were included. Studies were excluded if HIIT/SIT was combined with resistance training, supplementation, or ergogenic aids. All studies assessed muscle strength and/or muscle mass/size and/or ME, reported as pre/post intervention values or absolute/percentage change (means with standard deviations or standard error).

### 2.3. Information Sources and Search Strategy

Four online databases (Ovid Medline, Sport Discus, EMBASE, and CINAHL) were systematically searched for this review/meta-analysis (14 March 2023, 23 March 2025). The search strategy was based on including (“High-intensity interval training” OR “Sprint interval training” OR “High-intensity intermittent exercise” OR “Aerobic interval training” OR “SIT” OR “HIIT”) AND ((“Muscle strength” OR “Strength”) OR (“Muscle hypertrophy” OR “Hypertrophy” OR “Muscle growth”) OR (“Muscle endurance” OR “Strength endurance” OR “Muscle fatiguability” OR “Fatigue resistance”)). The full search strategy can be found in Table S1. A gray literature search and citation searching was performed using Google Scholar (14 March 2023, 24 March 2025).

### 2.4. Selection Process

Seven reviewers (L.W, J.ML, D. D, R. B, M.DF, M. A, J. L) applied the eligibility criteria and selected all studies for inclusion. All reviewers screened studies independently, and studies were screened in duplicate. One reviewer (L.W) screened all studies, and the other six reviewers (J.ML, D.D, R.B, M.DF, M.A, J. L) each screened approximately one sixth of the studies (randomly assigned). Researchers were blinded to each other’s decisions. Disagreements between individuals were resolved via a group discussion once all studies had passed through the given phase of screening (title and abstract or full text). Cohen’s kappa and percentage agreement were calculated via Covidence (Covidence systematic review software, Veritas Health Innovation, Melbourne, Australia. Available at www.covidence.org, Version 2) for both the abstract/title screening and full-text screening phases. For the abstract/title screening the average Cohen’s kappa and percentage agreement were 0.42 and 96.4%, respectively. A percentage agreement of 68.5% and an average Cohen’s kappa of 0.43 was observed for the full-text screening phase. Covidence (Covidence systematic review software, Veritas Health Innovation, Melbourne, Australia. Available at www.covidence.org, Version 2) was used for this review.

### 2.5. Data Collection Process Data Items

Study details, including author(s), participant characteristics (age, training status, number of participants), training prescription (duration of intervals, intensity of intervals, rest between intervals, and number of sets), mode of training (e.g., cycling, running, rowing, etc.), control group (yes/no), measure used to quantify strength, hypertrophy or ME, and main findings of the study, were extracted in duplicate and put into a custom data file (Microsoft Excel for Mac (Version 16.87)) and stratified by outcome (i.e., FFM, local muscle hypertrophy, strength, and ME). Disagreements between individuals were resolved via group discussion. Study investigators were contacted for unreported data/additional details when unreported data were deemed pertinent to the review/meta-analysis. When data were only provided in figures and investigators were unresponsive, WebPlotDigitizer (V4.6) was used to extract relevant data.

Acceptable measurement methods for strength outcomes included: 1-RM, isometric maximal volitional contraction (MVC), and isokinetic peak torque. For muscle mass/size, limb/segment/whole-body fat-free mass (assessed using dual-energy X-ray absorptiometry (DXA)), air displacement plethysmography (ADP), bioelectrical impedance (BIA)), muscle cross-sectional area (CSA), muscle thickness and muscle volume (assessed via ultrasonography or magnetic resonance imaging (MRI)), or muscle fiber CSA were included. Measures were accepted for ME at any load in absolute or relative terms or as muscle fatiguability in the form of maximum repetitions, work, or volume completed.

### 2.6. Risk of Bias Assessment

The Cochrane risk-of-bias 2 tool [34] was used to assess the risk of bias for the randomization process, deviation from intended interventions, missing outcome data, measurement of the outcome, selection of the reported result, and overall analysis. The Cochrane risk-of-bias 2 tool was chosen due to its rigor, widespread use within the field, and authors previous experience with this tool [35]. Four of the authors applied the risk-of-bias assessment (L.W, M.DF. J.ML, M.A). Each study was assessed by two independent reviewers (blinded to each other’s decisions). Disagreement was settled by a third reviewer.

### 2.7. Certainty of Evidence

The certainty of evidence was assessed via the GRADE (grading, recommendations, assessment, development, and evaluation) quality analysis framework for studies included in the meta-analysis (Tables S2–S4) [36]. As all studies included in the meta-analysis were randomized control trials, the evidence certainty was initially set as high (study design). Certainty was downgraded if >25% of studies were deemed as having a high risk of bias (risk of bias); if there was minimal overlap in confidence intervals or considerable heterogeneity (I^2^ > 50%) (inconsistency); if major discrepancies existed between participant demographics, training interventions, or measured outcomes or if indirect comparisons were made (indirectness); if confidence intervals exceeded 0.5 on either side of the standardized mean difference (SMD) (imprecision); and if publication bias was detected (if Egger’s test reached significance). Evidence was upgraded if there was a large effect (SMD > 0.8), plausible residual opposing confounding, and the presence of a dose response.

### 2.8. Data Synthesis and Analysis

A meta-analysis was performed when there were three or more randomized controlled trials comparing HIIT/SIT and a common comparator condition. Based on available data from included studies, three meta-analyses were performed. Post-intervention outcome means (SD) pooled between group comparisons (control as reference) were made with a Hedges G meta-analysis, using a random effects model (95% CI), with inverse variance weighting (restricted maximum likelihood estimation). A random effects model was chosen given expected heterogeneity across studies and that the target of inference extends beyond the samples contained within each study [37]. A Hartung–Knapp adjustment was made for small samples. Heterogeneity was assessed as the between-study variance (*τ*^2^) and proportion of variance attributable to between-study inconsistency (I^2^). A prediction interval was calculated to provide insight into the range of predicted treatment effect values on an individual level in a new study setting (after accounting for heterogeneity and within and between study variability) [38]. All post-intervention means were pooled together regardless of intervention length. Due to too few studies (n < 10), publication and small study bias were not formally explored. A ‘leave-one-out’ sensitivity analysis was performed to identify influential studies (see Figures S2, S4 and S6). All analyses were performed in R (version 4.3.2, R Core Team, Vienna, Austria) using the ‘meta’ package. After meta-analyses, the overall certainty of evidence was rated using the GRADE approach as described above (see Tables S1–S3) [36].

### 2.9. Post Hoc Protocol Deviations

Due to a lack of common comparator groups, for strength and muscle size/mass outcomes, additional post hoc analyses were performed. Weighted effect sizes and percentage change were calculated in Microsoft Excel (Microsoft Excel for Mac (Version 16.87)) via the formulas below [39,40]. (ES = effect size, n = study sample size, N = pooled sample size, Studypre = study pre-intervention mean, Studypost = study post-intervention mean, PooledSD = pre/post pooled standard deviation.) Confidence intervals were calculated using an alpha of 0.05.

$$WeightedES=\sum \frac{\left(n\times \frac{Studypre-Studypost}{PooledSD}\right)}{N}$$

$$Weighted\%∆=\sum \frac{\left(n\times \frac{Studypre-Studypost}{Studypre}\right)}{N}\times 100\%$$

## 3. Results

### 3.1. Study Selection

**Figure 1:**
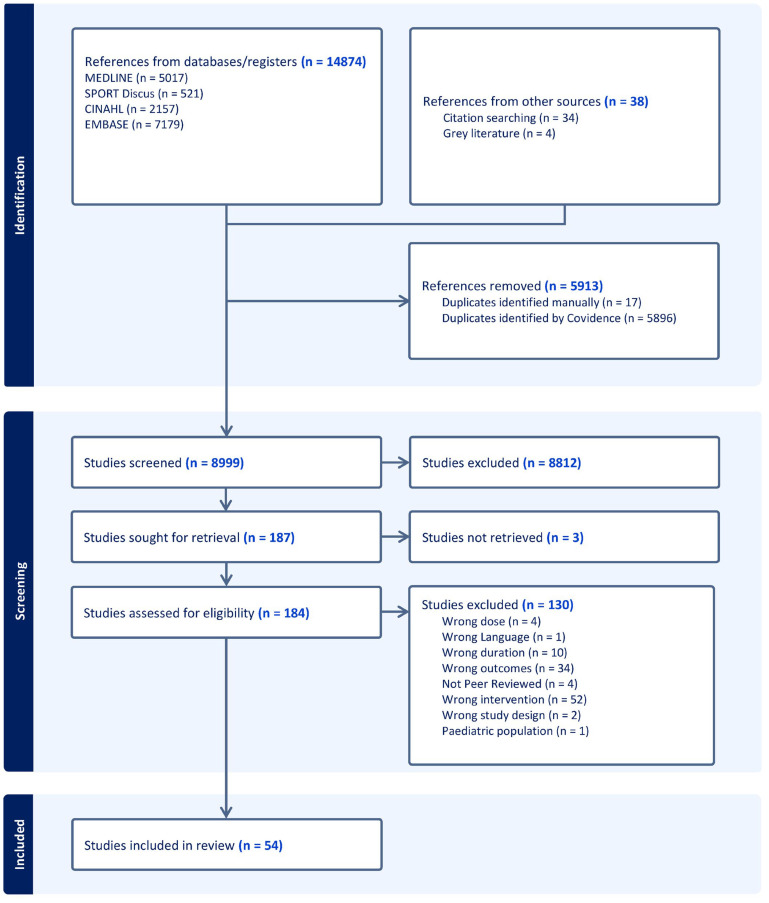
PRISMA flow diagram for study inclusion.

14,874 studies were retrieved from the initial database search along with 34 studies from citation searching and 4 studies from the gray literature. A total of 184 full-text articles were assessed for inclusion, with 54 studies ultimately included in this review (Figure 1).

### 3.2. Study Characteristics

Full study characteristics are included in Table 1, Table 2, Table 3 and Table 4. Of the 54 studies included, 32 studies (41 interventions) assessed FFM/skeletal muscle mass (Table 1), 19 studies (24 interventions) assessed local muscle mass/size or muscle fiber size (Table 2), 27 studies (37 interventions) measured muscle strength (Table 3), and 5 studies (8 interventions) assessed ME/fatiguability (Table 4). Of the 54 studies included in the review, 19 studies (35.2%) included male participants only, 10 studies (18.5%) included female participants only, and 25 studies (46.3%) included both males and females. The age of participants ranged from 18–80 years old. The training status of participants varied across studies, 16 recreationally active (29.6%), 18 sedentary (33.3%), 8 untrained (14.9%), 3 trained (5.6%), and 4 of unknown training status (7.4%). Some studies of note that initially appeared to meet the inclusion criteria were excluded due to RT being incorporated into the intervention groups’ warm up [41], the use of functional HIIT exercise interventions [42,43], and the duration of the intervention/intervals [44].

### 3.3. Risk of Bias

**Figure 2:**
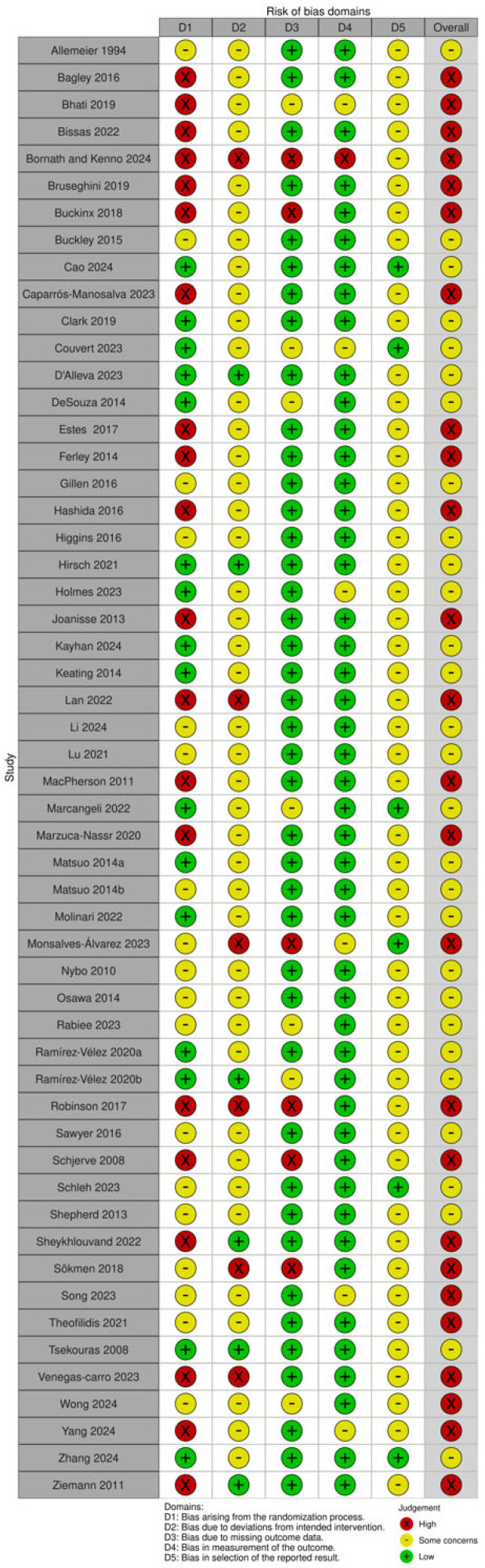
Assessment Tool [6,45,46,47,48,49,50,51,52,53,54,55,56,57,58,59,60,61,62,63,64,65,66,67,68,69,70,71,72,73,74,75,76,77,78,79,80,81,82,83,84,85,86,87,88,89,90,91,92,93,94,95,96,97,98,99]. Green, yellow, and red circles indicate a low risk of bias, some concerns for a risk of bis, or a high risk of bias, respectively, for the given domain. D1 (bias arising from the randomization process), D2 (bias due to deviations from intended intervention), D3 (bias due to missing outcome data), D4 (bias in measurement of the outcome), D5 (bias in selection of the reported result).

Twenty-five studies were assessed as having a high risk of bias, while twenty-nine were evaluated as having some concerns of bias (Figure 2).

### 3.4. Meta-Analysis Results

#### 3.4.1. FFM

**Figure 3:**
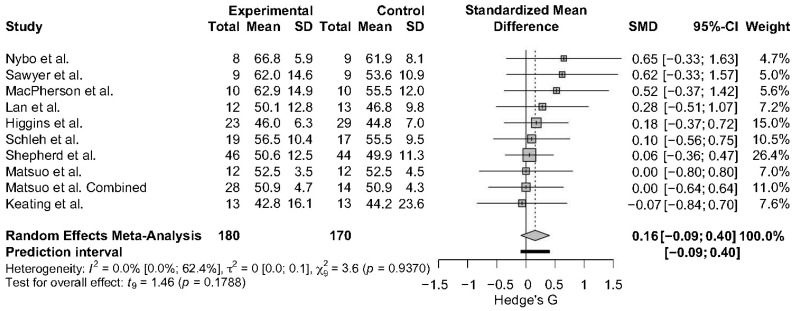
The Forest Plot for the meta-analysis between HIIT/SIT and MICT on FFM [55,56,59,62,63,65,70,71,72,81]. Gray boxes indicate study-specific estimates. Black lines through gray boxes represent 95% CIs. The gray diamond indicates overall pooled estimate. The thick black line indicates the predication interval.

A meta-analysis comparing 6 or more weeks of HITT/SIT to MICT was conducted on FFM (N = 10 studies, n = 333 participants). Seven studies used DXA [55,62,63,65,70,71,81] to assess FFM, while two used BIA [56,72], and another used ADP [59]. The analysis resulted in a standardized mean difference of 0.16 (95% CI: −0.09, 0.40), and little to no heterogeneity was detected: I^2^ =0.0% [95% CI; 0%; 62.4%] and *τ*^2^ = 0 [95% CI: 0.0; 0.1] (Figure 3). The leave-one-out analysis and Baujat Plot (Figure S2) suggests that Shepherd et al., 2013 [72], was the most influential on these results. The effect size varied minimally when studies were removed via the leave-one-out analysis with a range of SMD of 0.13, 0.19, indicating that these results are robust (Figure S3).

**Figure 4:**
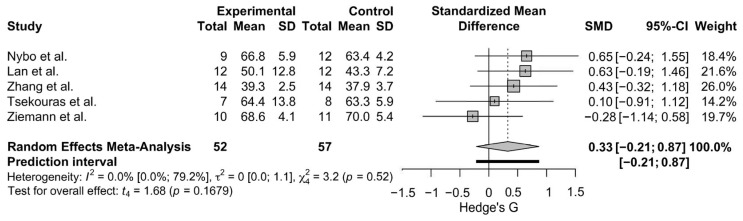
The Forest Plot for the meta-analysis between HIIT/SIT and CON on FFM [6,56,65,74,99]. Gray boxes indicate study-specific estimates. Black lines through gray boxes represent 95% CIs. The gray diamond indicates overall pooled estimate. The thick black line indicates the predication interval.

A meta-analysis comparing 6 or more weeks of HITT/SIT to CON was conducted on FFM (N = 5 studies, n = 109 participants). Two studies used DXA [74,100] to assess FFM, while three used BIA [6,56,75]. The analysis resulted in a standardized mean difference of 0.33 (95% CI: −0.21, 0.87), and little to no heterogeneity was detected: I^2^ = 0.0% [95% CI; 0%; 79.2%] and *τ*^2^ = 0 [95% CI: 0.0; 1.1] (Figure 4). The leave-one-out analysis and Baujat Plot (Figure S4) suggests that Ziemann et al., 2011 [6], was the most influential study on these results. The effect size varied when studies were removed via the leave-one-out analysis with a range of SMD of 0.24, 0.48 (Figure S5).

#### 3.4.2. Leg Press 1-RM Strength

**Figure 5:**
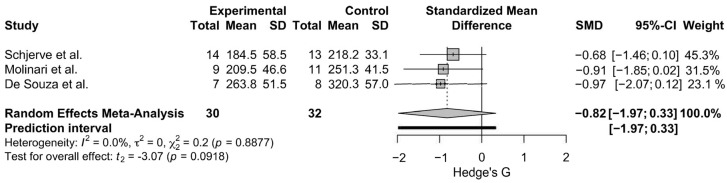
The Forest Plot for the meta-analysis between HIIT/SIT and RT on leg press 1-RM [78,92,93]. Gray boxes indicate study-specific estimates. Black lines through gray boxes represent 95% CIs. The gray diamond indicates overall pooled estimate. The thick black line indicates the predication interval.

A meta-analysis comparing 6 or more weeks of HITT/SIT to RT on leg press 1-RM strength (N = 3 studies, n = 62 participants) was conducted. The analysis resulted in a standardized mean difference of −0.82 (95% CI: −1.97, 0.33), and little to no heterogeneity was detected (I^2^ = 0.0%, *τ*^2^ = 0) (Figure 5). The leave-one-out analysis and Baujat Plot (Figure S6) suggests that Schjerve et al., 2008 [93], was the most influential study. The effect size varied when studies were removed via the leave-one-out analysis with a range of SMD of −0.94, −0.78 (Figure S7).

### 3.5. Weighted Effect Size and Percentage Change

#### 3.5.1. Weighted Effect Size and Percentage Change for Muscle Hypertrophy

Weighted effect sizes and percent changes were calculated for FFM, LLM, and quadriceps CSA (Table 5). The FFM calculation included 463 participants from 26 studies [6,45,46,47,48,49,50,51,54,55,56,58,59,60,61,62,63,64,66,70,71,72,73,74,75,81] and yielded a weighted effect size of 0.06 (−0.03, 0.15) and a weighted %∆ of 1.17 ± 1.64%. The LLM calculation included 159 participants from seven studies [51,54,60,61,69,89,101], a weighted effect size of 0.04 (0.02, 0.07) and a weighted %∆ of 0.61 ± 2.36% was determined. Lastly, an effect size of 0.36 (0.34, 0.37) and a weighted %∆ 4.72 ± 1.35% of was observed for quadriceps CSA. This calculation included 71 participants from four studies [46,66,78,101]

#### 3.5.2. Weighted Effect Size and Percentage Change for Muscle Strength

Weighted effect sizes and percent changes were calculated for three strength outcomes: leg press 1-RM, isokinetic knee extension at 60°/s, and isometric knee extension at 90° (Table 6). The leg press 1-RM calculation included 41 participants from four studies [78,92,93,96]. A weighted %∆ 3.45 ± 2.19% and weighted effect size of 0.16 (0.13, 0.19) were determined. The calculation for isokinetic knee extension at 60°/s involved 163 participants across seven studies [46,48,73,80,91,95,101], a weighted effect size of 0.01 (−0.02, 0.04) and a weighted %∆ of 0.35 ± 4.88% was yielded. Lastly, an effect size of 0.19 (0.15, 0.22) and a weighted %∆ of 4.94 ± 5.82% was calculated for isometric knee extension at 90° of knee flexion. This calculation involved 108 participants from five studies [45,46,89,97,101].

## 4. Discussion

The main findings of the present study indicate that HIIT/SIT may induce greater gains in FFM relative to MICT and non-exercise control groups. The results also suggest that RT is likely superior to HIIT/SIT regarding increases in leg press 1-RM strength. However, these findings should be interpreted with caution due to the small samples, imprecision of the pooled estimates (i.e., wide 95% CIs), 95% CIs that intersected zero for all three meta-analyses, and the certainty of evidence being classified as “low” for all analyses (Tables S2–S4). Pre–post effect sizes also suggest that participants who underwent HIIT/SIT interventions incurred small to moderate gains in quadriceps CSA and small improvements in isometric knee extension (90°) and leg press 1-RM strength, while minute to no change was observed in FFM, LLM, and isokinetic knee extension (60°/s) (Table 4 and Table 5). These observations suggest the potential benefits of HIIT/SIT for muscle strength and muscle size; however, further investigations with non-exercise control groups are required prior to the formation of firm conclusions on this topic.

### 4.1. Hypertrophy

Meta-analyses comparing HIIT/SIT to non-exercise controls and MICT indicate non-significant small effect sizes in favor of HIIT/SIT for FFM. Further, evidence from the systematic review indicates that HIIT/SIT likely has a very small effect on FFM, as a pre–post weighted effect size of 0.07 CI (−0.05, 0.08) was calculated (n = 463). These results are in agreement with that of Sultana et al., 2019 [19]. However, HIIT/SIT may be more effective at increasing localized muscle size when assessed via MRI. All five interventions that assessed pre–post changes in quadriceps CSA observed increases from baseline following HIIT/SIT (Table 5), and a small–moderate pre–post weighted effect size of 0.37; 95% CI (0.31, 0.44) was determined for this outcome (n = 71). This effect size is lesser but similar to that determined by Schoenfeld and colleagues [23] for the effects of low-load (0.42; 95% CI (0.23, 0.60)) and high-load RT (0.53; 95% CI (0.30, 0.76)) on muscle hypertrophy. No meta-analysis was conducted on quadriceps CSA for HIIT/SIT due to a lack of common comparator groups, limiting the conclusions that can be made for this outcome relative to RT or to non-exercise controls.

During a RT set, initially recruited motor unit fatigue over subsequent repetitions due to the accumulation of metabolites resulting in increased motor unit recruitment, with task failure resulting when further increases in motor units or the firing rate are not possible [102,103]. Training in proximity to failure and achieving sufficient training volume are key determinants of training-induced muscle hypertrophy [12,23]. Although time efficient, a substantial volume of mechanical work is typically achieved during a HIIT/SIT session [4]. HIIT occurs at a constant high intensity (80–95% VO_2_max), while SIT is completed at maximal or supramaximal intensity and is characterized by a steep decline in power output over time [14], while neither typically result in momentary task failure. The lack of momentary failure achieved during HIIT/SIT may partially explain the lower calculated effect sizes for HIIT/SIT relative to that of low-load and high-load RT from Schoenfeld et al., 2017 [23]. However, this fails to explain why much smaller magnitudes of effect sizes were determined for FFM and leg lean mass relative to MRI derived quadriceps CSA.

MRI is considered the gold standard of hypertrophic assessment as near perfect correlations have been established between MRI and cadaveric measures of muscle size and volume (r = 0.99) [104,105]. When compared to MRI, ultrasound and DXA both show strong cross-sectional correlations of r = 0.99, 0.89, respectively [106,107]. ADP and BIA display a strong correlation with DXA (r = 0.94 and 0.84–0.89), respectively, when comparing cross-sectional measurements [108]. However, a correlation of only 0.49 between DXA and MRI was observed after a 10-week RT program, exemplifying lower sensitivity of DXA to detect changes in muscle size across intervention studies [108]. BIA also performs poorly compared to DXA when measuring changes in lean body mass over time [109], as a 12-week weight loss study found a correlation of only 0.24 between DXA and BIA in measurement of FFM [109]. Further, BIA, DXA, and ADP measure FFM, not muscle mass. Therefore, limited value can be placed on FFM as a proxy for local muscle hypertrophy. For example, if a 100 kg person with a quadriceps muscle volume of 5000 cm^3^, a leg lean mass of 10 kg, and a FFM of 60 kg completed a quadriceps dominant training protocol for 12 weeks and increased their quadriceps muscle volume by 5% (250 cm^3^), this would be realized by only a ~2.65% gain in leg lean mass (265 g) and only a ~0.44% change in terms of FFM (assuming no other FFM changes and a muscle density of 1.0597 g/cm^3^). Furthermore, the technical error of the measurement has been estimated at ~2.5% for DXA-derived thigh mass [110] and only 1.1% for MRI-based thigh muscle volume [111]. Therefore, it is unsurprising that changes are more frequently observed via MRI measures of localized hypertrophy compared to DXA even within the same intervention group [66]. This phenomenon is demonstrated in the pre–post effect sizes calculated in Table 4.

### 4.2. Strength

The results from the meta-analysis comparing HIIT/SIT to RT on leg press 1-RM yielded a large but non-significant effect size in favor of RT. The findings of this meta-analysis should be interpreted cautiously given its small base of only three studies and 62 participants. However, all three studies observed a moderate–large effect in favor of RT suggesting that RT is likely superior to HIIT/SIT at increasing leg press 1-RM. Though, it should be noted that HIIT/SIT may induce a small increase in strength, as a pre–post weighted percentage change of 3.45 ± 2.19% and 4.94 ± 5.82% was determined for leg press 1-RM and isometric knee extension (90°), respectively (Table 5).

Traditional RT loads range from 30 to 90% 1-RM, with higher-load RT (>70% 1-RM) typically inducing greater gains in muscle strength than low-load RT (<60% 1-RM) [23]. HIIT/SIT induces high levels of perceived exertion, but peak forces during cycling-based HIIT/SIT are estimated to be only ~10–25% 1-RM [27,28]. Findings from the studies presented in this review reveal that HIIT/SIT does not induce consistent gains in muscle strength. This can be best explained by the principal of specificity, whereby the more similar a training stimuli is to the strength assessment in terms of load and modality, the larger the resulting improvement in task performance [23,112,113]. Recently, Pallares et al., 2025, demonstrated similar strength gains between 10-week cycling- and squat-based RT training, both occurring at 70% maximal dynamic force [114]. Therefore, the most plausible reasoning for HIIT/SIT not inducing gains in strength in most studies is due to insufficient loading rather than other dissimilarities between cycling and RT. Developing HIIT/SIT interventions, which induce forces above 60% of peak force, potentially through a reduction in cadence, would likely result in greater strength gains.

### 4.3. Muscle Endurance

In the present review, five studies were included that assessed ME or muscle fatiguability. Bornath and Kenno, 2022 [86], utilized a battle rope-based SIT intervention over 6 weeks in recreationally active younger adults. Both the male and female groups in this study improved their number of push-ups and sit-ups completed to failure and isometric shoulder extension and flexion strength relative to baseline. Similar results were observed by Cao et al., 2024 [88], who observed an increase in the number of push-ups completed following 12 weeks of running-based SIT. In contrast, Buckley et al., 2015 [87], found no difference in squat endurance at 70% of pre-training 1-RM after 8 weeks of a rowing-based HIIT intervention. The disparity between Buckley et al., 2015 [87], and Bornath and Kenno, 2022 [86], regarding ME is likley explained by the chosen load of the test used in each study and the relative load used during training [115].

Two other HIIT/SIT Intervention studies included in the review assesed muscle fatiguability [73,77]. In these studies, a maximal knee extension exercise was performed at 120°/s and 60°/s, respectively, and a fatigue index was used to assess the drop-off in force/work over 60 repetitions in the case of Bagley et al., 2016 [77], and until less than 50% of max work was maintained in Theofilidis et al., 2021 [73]. In both studies, muscle fatuigability was improved following HIIT/SIT, exhibiting that HIIT/SIT may improve fatigue resistance.

Due to a lack of similarity between ME measurements and common comparator groups, no meta-analyses or effect size calculations were possible for ME limiting the conclusions that can be drawn regarding this outcome. However, the current evidence suggests that HIIT/SIT likely improves low-load absolute ME but not high-load absolute ME or relative ME, as the latter are primarily governed by muscle strength [29].

### 4.4. Limitations

The findings of the present systematic review are limited due to a lack of non-exercise control groups or common comparator interventions and a lack of standardization in methods of strength, muscle mass/size, and ME assessment. Furthermore, no inferences can be made regarding the role of HIIT/SIT on strength, muscle hypertrophy, and ME in the presence of nutritional/ergogenic supplementation or acute and chronic conditions, as studies including supplementation and participants with acute or chronic ailments were excluded.

### 4.5. Conclusions and Future Directions

This review demonstrates that HIIT/SIT may induce slightly greater gains than MICT and non-exercise controls for FFM, while RT is likely superior to HIIT/SIT regarding 1-RM leg press strength. However, the certainty of evidence is low, and 95% CIs intersect zero for all three analyses. Pre–post effect sizes suggest that HIIT/SIT may induce small to moderate increases in quadriceps CSA and small increases in isometric and 1-RM strength, but no firm conclusions can be drawn for localized hypertrophy, strength, or ME from this review. Further research using control groups and validated methodologies to assess muscle strength, muscle mass/size, and ME are required to increase the level of certainty and clarity in these conclusions.

## Figures and Tables

**Table 1:** An overview of the main characteristics of included studies measuring FFM. SIT (sprint interval training), HIIT (high-intensity interval training), S (seconds), Min (minutes), (LV (low volume), HV (high volume), Per (periodized), Trad (traditional), HR max (heart rate maximum), Watt-peak (peak wattage in a ramp test), VO_2_max/peak (maximal/peak oxygen consumption), MAS (maximum aerobic speed), FFM (fat-free mass), SMM (skeletal muscle mass) DXA (dual X-ray absorptiometry), BIA (bioelectrical impendence analysis), ADP (air displacement plethysmography), ↑ indicates a significant increase from baseline, ↔︎ indicates no change from baseline.

Study	Sample Size	Training Status/Age	Group Name	Protocol	Modality	Duration (Weeks)	Hypertrophy Outcome	Method(s) of Assessment
Bhati 2019 [45]	17	Sedentary, Young adults	HIIT (LV)	1 × (4 min at 85–95% of HR max, 3 min at 70% HR max)	Running	6	FFM ↑	BIA
Bhati 2019 [45]	15	Sedentary, Younger Adults	HIIT (HV)	4 × (4 min at 85–95% of HR max, 3 min at 70% HR max)	Running	6	FF︎M ↔︎	BIA
Bruseghini 2019 [46]	12	Recreational, Older Adults	HIIT	7 × (2 min at 85–95% VO_2_max, 2 min rest)	Cycling	8	FFM ↔︎	DXA
Buckinx 2018 [47]	30	Sedentary Obese, Older Adults	HIIT	10 × (30 s at 80–85% HR max, 90 s at 65% HR max)	Elliptical	12	FFM ↑	DXA
Clark 2019 [48]	8	Sedentary Obese, Adults	HIIT (Per)	6–10 × (20 s–2 min at 65–110% peak power, 1–2 min rest)	Cycling	6	FFM ↔︎	ADP
Clark 2019 [48]	9	Sedentary Obese, Adults	HIIT (Trad)	10 × (1 min at 70–85% peak power, 60 s rest)	Cycling	6	FFM ↑	ADP
Couvert 2024 [49]	8	TS Unknown, Obese/Overweight, Adults	HIIT	9 × (45 s at 80–85% HR max, 90 s active recovery)	Running	12	FFM ↔︎	DXA
Couvert 2024 [49]	8	TS Unknown, Obese/Overweight, Adults	HIIT	10 × (45 s at 80–85% HR max, 90 s active recovery)	Cycling	12	FFM ↔︎	DXA
D’Alleva 2023 [50]	16	Sedentary, Obese, Adults	HIIT	5–7 × (2 min at 95% VO_2_peak, 1 min at 50% VO_2_peak)	Running	12	FFM ↔︎	BIA
Gillen 2016 [51]	8	Sedentary Obese, Adults	HIIT (Fed)	10 × (1 min at 90% HR max, 1 min at 50 W)	Cycling	6	FFM ↔︎	DXA
Gillen 2016 [51]	8	Sedentary Obese, Adults	HIIT (Fasted)	10 × (1 min at 90% HR max, 1 min at 50 W)	Cycling	6	FFM ↔︎	DXA
Higgins 2016 [52]	23	Sedentary Obese, Younger Adults	SIT	4–7 × (30 s at maximal effort, 4 min of active rest)	Cycling	6	FFM ↔︎	DXA
Hirsch 2021 [53]	19	Untrained Obese, Adults	HIIT	6–10 × (1 min at 90% watt-max, 60 s rest)	Cycling	8	FFM ↑	DXA
Holmes 2023 [54]	15	Recreational, Younger Adults	HIIT	30 min (20–80 s at unknown intensity, 10–60 s rest)	Cycling	8	FFM ↑	DXA
Keating 2014 [55]	13	Sedentary, Adults	HIIT	4–6 × (30–60 s at 120% VO_2_peak, 2–3 min at 30 W)	Cycling	12	FFM ↔︎	DXA
Lan 2022 [56]	12	Sedentary, Younger Adults	HIIT	4 × (4 min at 85–95% HR max, 3 min at 64–76% HR max)	Running	8	FFM ↔︎, SMM ↔︎	BIA
Li 2024 [57]	13	Untrained, Older Adults	HIIT	5 × (3 min at 85% HR max, 3 min at 55% HR max)	Walking	8	SMM ↑	BIA
Lu 2021 [58]	10	Untrained, Younger Adults	SIT	4 × (30 s at max effort, 30 s rest	Running	12	FFM ↑	BIA
MacPherson 2011 [59]	10	Recreational, Younger Adults	SIT	4–6 × (30 s at max effort, 4 min active rest)	Running	6	FFM ↑	ADP
Marcangeli 2022 [60]	45	Sedentary Obese, Older Adults	HIIT	10 × (30 s at 80–85% HR max, 90 s at 65% HR max)	Elliptical	12	FFM ↑	DXA
Marzuca-Nassr 2020 [61]	10	Sedentary, Younger Adults	HIIT	10 × (1 min at 90% HR max, 2 min rest)	Cycling	12	FFM ↔︎	DXA
Marzuca-Nassr 2020 [61]	10	Sedentary, Older Adults	HIIT	10 × (1 min at 90% HR max, 2 min rest)	Cycling	12	FFM ↔︎	DXA
Matsuo 2014a [62]	12	Sedentary, Adults	HIIT	3 × (3 min at 80–85% VO_2_max, 2 min at 50% VO_2_max)	Cycling	8	FFM ↔︎	DXA
Matsuo 2014b [63]	14	Sedentary, Adults	SIT	7 × (30 s at 120% VO_2_max, 15 s rest)	Cycling	8	FFM ↑	DXA
Matsuo 2014b [63]	14	Sedentary, Adults	HIIT	3 × (3 min 80–90% VO_2_max, 2 min at 50% VO_2_max)	Cycling	8	FFM ↑	DXA
Monsalves-Álvarez [64]	11	TS Unknown, Overweight/Obese Adults	HIIT	10 × (1 min at 85–90% HR max, 1 min at 50 W)	Cycling	12	FFM ↔︎	DXA
Nybo 2010 [65]	8	Untrained, Adults	HIIT	5 × (2 min at 95% HR max, 1 min rest)	Running	12	FFM ↔︎	DXA
Osawa 2014 [66]	7	Untrained, Adults	HIIT (Leg)	8–12 × (1 min at >90% watt-peak, 60 s active rest)	Cycling	16	FFM ↔︎	DXA
Osawa 2014 [66]	5	Untrained, Adults	HIIT (Arm/Leg)	8–12 × (60 s at >90% watt-peak, 60 s active rest) (half arm, half leg)	Cycling/Arm Cycling	16	FFM ↔︎	DXA
Rabbiee 2023 [67]	9	Sedentary, Obese/Overweight, Adults	HIIT	4–6 × (1 min at 90–95% HR max, 1 min rest)	Cycling	8	SMM ↔︎	BIA
Robinson 2017 [68]	14	Untrained, Younger Adults	HIIT (Young)	4 × (4 min at >90% of VO_2_max, 3 min at 0 W)	Cycling	12	FFM ↑	DXA
Robinson 2017 [68]	9	Untrained, Older Adults	HIIT (Old)	4 × (4 min at >90% of VO_2_max, 3 min at 0 W)	Cycling	12	FFM ↑	DXA
Ramirez-Velez 2020a [69]	14	Sedentary, Adults	HIIT	4 × (4 min at 85–95% HR max, 4 min at 65% HR max) week 3–12 max) week 3–12	Run/Walk with Incline	12	FFM ↑	DXA
Sawyer 2016 [70]	9	TS Unknown Obese, Adults	HIIT	10 × (1 min at 90–95% of HR max, 1 min at 25–50 W)	Cycling	8	FFM ↔︎	DXA
Schleh 2023 [71]	19	Sedentary, Obese, Adults	HIIT	10 × (1 min at 90% HR max, 1 min at 65% HR max)	Cycling/Rowing/ Running/ Elliptical	12	FFM ↔︎	DXA
Shepherd 2013 [72]	42	Sedentary, Adults	HIIT	4–12 × (15–60 s at >90% HR max, 45–120 s active rest)	Cycling	10	FFM ↔︎	BIA
Theofilidis 2021 [73]	7	Recreational, Adults	HIIT (Up)	10 × (30 s at 90% MAS +10% grade, 60 s rest)	Running	8	FFM ↔︎	BIA
Theofilidis 2021 [73]	7	Recreational, Adults	HIIT (Down)	10 × (30 s at 90% MAS −10% grade, 60 s rest)	Running	8	FFM ↔︎	BIA
Tsekouras 2008 [74]	7	Sedentary, Younger Adults	HIIT	4 × (4 min at 90% VO_2_peak, 4 min at 60% VO_2_peak)	Running	8	FFM ↔︎	DXA
Zhang 2024 [75]	14	Sedentary, Younger Adults	HIIT	2–3 × (12–15 × 30 s at max effort, 10 s rest)	Cycling	8	FFM ↔︎	BIA
Ziemann 2011 [6]	10	Recreational, Younger Adults	HIIT	6 × (90 s at 80% VO_2_max, 3 min rest)	Cycling	6	FF︎M ↔︎	BIA

**Table 2:** An overview of the main characteristics of included studies measuring localized muscle hypertrophy. SIT (sprint interval training), HIIT (high-intensity interval training), S (seconds), Min (minutes), HR max (heart rate maximum), Watt-peak (peak wattage in a ramp test), VO_2_max/peak (maximal/peak oxygen consumption), MAS (maximum aerobic speed), CSA (cross-sectional area), LLM (leg lean mass), Quad (quadriceps), RF (rectus femoris), Thigh LM (thigh lean mass), VL (vastus lateralis), ALM (arm lean mass), LBLM (lower body lean mass), UBLM (upper body lean mass), Psoas (psoas major), Alab (anterolateral abdominal), Trunk LM (trunk lean mass), DXA (dual X-ray absorptiometry), BIA (bioelectrical impendence analysis), MRI (magnetic resonance imaging), ↑ indicates a significant increase from baseline, ↔︎ indicates no change from baseline, ↓ indicates a significant decrease from baseline.

Study	Sample Size	Training Status/Age	Group Name	Protocol	Modality	Duration (Weeks)	Hypertrophy Outcome	Method(s) of Assessment
Allemeier 1994 [76]	11	Untrained, Young Adults	SIT	3 × (30 s Wingate, 20 min rest)	Cycling	6	Type 1 CSA ↔︎, Type 2a CSA ↔︎, Type 2× CSA ↔︎	Histology
Bagley 2016 [77]	15	Recreational, Adults	SIT (Female)	4 × (20 s at 175–200% VO_2_peak, 2 min rest)	Cycling	12	LLM ↔︎, Thigh Lean Mass ↔︎, Quad CSA ↑	DXA, MRI
Bagley 2016 [77]	16	Recreational, Adults	SIT (Male)	4 × (20 s at 175–200% VO_2_peak, 2 min rest)	Cycling	12	LLM ↔︎,Thigh Lean Mass ↔︎, Quad CSA ↑	DXA, MRI
Bruseghini 2019 [46]	12	Recreational, Older Adults	HIIT	7 × (2 min at 85–95% VO_2_max, 2 min rest)	Cycling	8	Quad CSA ↑, Quad Volume ↑	MRI
De Souza 2014 [78]	8	Recreational, Adults	HIIT	15–20 × (60 s at VO_2_peak, rest time/intensity not reported)	Running	8	Thigh CSA ↔︎ Type 1 CSA ↔︎, Type 2a CSA ↔︎, Type 2× CSA ↔︎	MRI, Histology
Estes 2017 [79]	12	Recreational, Younger Adults	HIIT	4 × (4 min at 90–95% HR max, 3 min rest)	Running	10	VL CSA ↑	Ultrasound
Gillen 2016 [51]	8	Sedentary Obese, Adults	HIIT (Fed)	10 × (1 min at 90% HR max, 1 min at 50 W)	Cycling	6	LLM ↑, Gynoid LM ↑	DXA
Gillen 2016 [51]	8	Sedentary Obese, Adults	HIIT (Fasted)	10 × (1 min at 90% HR max, 1 min at 50 W)	Cycling	6	LLM ↑, Gynoid LM ↑	DXA
Hashida 2016 [80]	15	Recreational, Younger Adults	HIIT	5 × (2 min at 60–90% VO_2_peak, 2 min at 40% VO_2_peak)	Cycling	6	RF Muscle Thickness ↔︎	Ultrasound
Higgins 2016 [81]	23	Sedentary Obese, Younger Adults	SIT	4–7 × (30 s at maximal effort, 4 min of active rest)	Cycling	6	LLM ↑	DXA
Hirsch 2021 [53]	19	Untrained Obese, Adults	HIIT	6–10 × (1 min at 90%-watt-max, 60 s rest)	Cycling	8	Thigh LM ↑, VL CSA ↑, VL Volume ↑	DXA, Ultrasound
Holmes 2023 [54]	15	Recreational, Younger Adults	HIIT	30 min (20–80 s at unknown intensity, 10–60 s rest)	Cycling	8	LLM ↔︎, ALM ↔︎, VL CSA ↔︎	DXA, Ultrasound
Joanisse 2013 [82]	15	Sedentary, Adults	HIIT	10 × (1 min at 90% HR max, 1 min at 50 W or passive rest)	Cycling	6	Type 1 CSA ↔︎, Type 2 CSA ↔︎, Hybrid CSA ↔︎	Histology
Marcangeli 2022 [60]	45	Sedentary Obese, Older Adults	HIIT	10 × (30 s at 80–85% HR max, 90 s at 65% HR max)	Elliptical	12	LLM ↑, ALM ↔︎	DXA
Marzuca-Nassr 2020 [61]	10	Sedentary, Younger Adults	HIIT	10 × (1 min at 90% HR max, 2 min rest)	Cycling	12	LLM ↑	DXA
Marzuca-Nassr 2020 [67]	10	Sedentary, Older Adults	HIIT	10 × (1 min at 90% HR max, 2 min rest)	Cycling	12	LLM ↔︎	DXA
Monsalves-Álvarez 2023 [64]	11	TS Unknown, Overweight/Obese Adults	HIIT	10 × (1 min at 85–90% HR max, 1 min at 50 W)	Cycling	12	RF CSA ↔︎	Ultrasound
Nybo 2010 [65]	8	Untrained, Adults	HIIT	5 × (2 min at 95% HR max, 1 min rest)	Running	12	LLM ↔︎	DXA
Osawa 2014 [66]	7	Untrained, Adults	HIIT (Leg)	8–12 × (1 min at >90% watt-peak, 60 s active rest)	Cycling	16	Quad CSA ↑, Hamstring CSA ↔︎, LBLM ↔︎, UBLM, ↔︎ Psoas CSA ↔︎, Alab CSA ↔︎, Spinal CSA ↔︎	DXA, MRI
Osawa 2014 [66]	5	Untrained, Adults	HIIT (Arm/Leg)	8–12 × (60 s at >90% watt-peak, 60 s active rest) (half arm, half leg)	Cycling/Arm Cycling	16	Quad CSA ↑, Hamstring CSA LBLM ↔︎, UBLM ↔︎, Psoas CSA ↑, Alab CSA ↔︎, Spinal CSA ↔︎	DXA, MRI
Ramirez-Velez 2020b [83]	11	Sedentary, Adults	HIIT	4 × (4 min at 85–95% HR max, 4 min at 65% HR max) week 3–12	Running	12	LLM ↔︎, ALM ↔︎, Trunk LM ↔︎	BIA
Theofilidis 2021 [73]	7	Recreational, Adults	HIIT (Uphill)	10 × (30 s at 90% MAS +10% grade, 60 s rest)	Hill Running	8	VL Thickness↓, VL Length ↓	BIA, Ultrasound
Theofilidis 2021 [73]	7	Recreational, Adults	HIIT (Downhill)	10 × (30 s at 90% MAS −10% grade, 60 s rest)	Running	8	VL Thickness↓, VL Length ↓	BIA, Ultrasound
Yang 2024 [84]	261	Sedentary, Younger Adults	HIIT	4 × (4 min at 80–90% VO_2_max, 3 min recovery at 50–55% VO_2_max) week 5–12	Running	12	RF Muscle Thickness ↑	Ultrasound

**Table 3:** An overview of the main characteristics of included studies measuring strength outcomes. SIT (sprint interval training), HIIT (high-intensity interval training), S (seconds), Min (minutes), m (meters), W (Watts), RPE (rating of perceived exertion), T max (time to exhaustion), V max (maximum velocity), LV (low volume), HV (high volume), Per (periodized), Trad (traditional), HR max (heart rate maximum), VO_2_max/peak (maximal/peak oxygen consumption), MAS (maximum aerobic speed), ISOM (isometric), ISOK (isokinetic), 1-RM (1 repetition maximum), PF (plantar flexion), ↑ indicates a significant increase from baseline, ↔︎ indicates no change from baseline, ↓ indicates a significant decrease from baseline.

Author	Sample Size	Training Status/Age	Group Name	Protocol	Modality	Duration (Weeks)	Strength Outcome
Bagley 2016 [77]	15	Recreational, Adults	SIT (Female)	4 × (20 s at 175–200% VO_2_peak, 2 min rest)	Cycling	12	ISOM Knee Extension (90°) ↔︎, ISOK Knee Extension (60°/s) ↔︎, ISOK Knee Extension (120°/s) ↔︎, ISOK Knee Extension (180°/s) ↔︎, ISOK Knee Extension (240°/s)
Bagley 2016 [77]	16	Recreational, Adults	SIT (Male)	4 × (20 s at 175–200% VO_2_peak, 2 min rest)	Cycling	12	ISOM Knee Extension (90°) ↔︎, ISOK Knee Extension (60°/s) ↔︎, ISOK Knee Extension (120°/s) ↔︎, ISOK Knee Extension (180°/s) ↔︎, ISOK Knee Extension (240°/s) ↔︎
Bhati 2019 [45]	17	Sedentary, Younger Adults	HIIT (LV)	1 × (4 min at 85–95% of HR max, 3 min at 70% HR max)	Running	6	ISOM Knee Extension (90°) ↔︎
Bhati 2019 [45]	15	Sedentary, Younger Adults	HIIT (HV)	4 × (4 min at 85–95% HR max, 3 min at 70% HR max)	Running	6	ISOM Knee Extension (90°) ↔︎
Bissas 2022 [85]	14	Recreational, Younger Adults	SIT (Up/Down)	6 × (80 m at max effort, 4–6 min rest)	Running	6	ISOM Knee Extension (107°) ↑, ISOM Knee Flexion (107°) ↔︎
Bissas 2022 [85]	7	Recreational, Younger Adults	SIT	6 × (80 m at max effort, 4–6 min rest)	Running	6	ISOM Knee Extension (107°) ↔︎, ISOM Knee Flexion (107°) ↔︎
Bornath and Kenno 2022 [86]	15	Recreational, Younger Adults	SIT (Female)	10 × (30 s at max effort, 60 s rest)	Battle Ropes	6	ISOM Shoulder Flexion (90°) ↑, ISOM Shoulder Extension (90°) ↑
Bornath and Kenno 2022 [86]	18	Recreational, Younger Adults	SIT (Male)	10 × (30 s at max effort, 60 s rest)	Battle Ropes	6	ISOM Shoulder Flexion (90°) ↑, ISOM Shoulder Extension (90°) ↑
Bruseghini 2019 [46]	12	Recreational, Older Adults	HIIT	7 × (2 min at 85–95% VO_2_max, 2 min rest)	Cycling	8	ISOM Knee Extension (90°) ↔︎, ISOM Knee Extension (60°) ↑, Eccentric ISOK Knee Extension (60°/s) ↔︎, Eccentric ISOK Knee Extension (120°/s) ↔︎, ISOK Knee Extension (60°/s) ↔︎, ISOK Knee Extension (120°/s) ↔︎
Buckinx 2018 [47]	30	Sedentary Obese, Older Adults	SIT	10 × (30 s at 80–85% HR max, 90 s at 65% HR max)	Elliptical	12	ISOM Knee Extension (135°) ↔︎
Buckley 2015 [87]	14	Recreational, Younger Adults	HIIT	6 × (1 min at RPE of 9–10/10, 3 min rest)	Rowing	6	Squat 1-RM ↔︎, Deadlift 1-RM ↔︎
Cao 2024 [88]	12	Sedentary, Younger Adults	SIT	4 × (4 × 30 s at 100–120% MAS, 30 s rest at 50% MAS)	Running	12	Back Extension Force ↑, Hand grip Strength ↑
Clark 2019 [48]	8	Sedentary Obese, Adults	HIIT (Per)	6–10 × (20 s–2 min at 65–110% peak power, 1–2 min rest)	Cycling	6	ISOK Knee Extension (60°/s) ↔︎, ISOK Knee Flexion (60°/s) ↔︎
Clark 2019 [48]	9	Sedentary Obese, Adults	HIIT (Trad)	10 × (1 min at 70–85% peak power, 60 s rest)	Cycling	6	ISOK Knee Extension (60°/s) ↔︎, ISOK Knee Flexion (60°/s) ↔︎
Caparrós-Manosalva 2023 [89]	9	Untrained, Younger Adults	HIIT(Young)	10 × (1 min at 90% HR max, 2 min rest)	Cycling	12	ISOM Knee Extension (90°) ↑
Caparrós-Manosalva 2023 [89]	9	Untrained, Older Adults	HIIT (Old)	10 × (1 min at 90% HR max, 2 min rest)	Cycling	12	ISOM Knee Extension (90°) ↑
De Souza 2014 [78]	8	Recreational, Younger Adults	HIIT	15–20 × (60 s at VO_2_peak, rest time/intensity not reported)	Running	8	Leg Press 1RM ↔︎
Ferley 2014 [90]	12	Endurance Trained, Younger Adults	SIT (Flat)	4–6 × (60% T max at V max, time to 65% HR max)	Running	6	ISOK Knee Flexion (90°/s) ↔︎, ISOK Knee Flexion (180°/s) ↑, ISOK Knee Flexion (300°/s) ↑
Ferley 2014 [90]	12	Endurance Trained, Younger Adults	SIT (Up)	10–14 × (30 s at V max, time to 65% HR max)	Running	6	ISOK Knee Flexion (90°/s) ↔︎, ISOK Knee Flexion (180°/s) ↑, ISOK Knee Flexion (300°/s) ↑
Hashida 2021 [80]	15	Recreational, Younger Adults	HIIT	5 × (2 min at 60–90% VO_2_peak, 2 min 40% VO_2_peak)	Cycling	6	ISOK Knee Extension (60°/s) ↔︎
Holmes 2023 [54]	15	Recreational, Younger Adults	HIIT	30 min (20–80 s at unknown intensity, 10–60 s rest)	Cycling	8	ISOK Knee Extension (60°/s) ↔︎, PF Extension (60°/s) ↔︎
Kayhan 2024 [91]	9	Recreational, Younger Adults	HIIT (Short rest)	2 × 300 m, 2 × 300 m, 2 × 400 m, 2 × 300, 2 × 200, all at 85% HRR, rest until 45% HRR	Running	8	ISOK Knee Extension (60°/s) ↔︎, ISOK Knee Flexion (60°/s) ↔︎, ISOK Knee Extension (240°/s) ↔︎, ISOK Knee Flexion (240°/s) ↔︎
Kayhan 2024 [91]	10	Recreational, Younger Adults	HIIT (Long rest)	2 × 300 m, 2 × 300 m, 2 × 400 m, 2 × 300, 2 × 200, all at 85% HRR, rest until 35% HRR	Running	8	ISOK Knee Extension (60°/s) ↔︎, ISOK Knee Flexion (60°/s) ↔︎, ISOK Knee Extension (240°/s) ↔︎, ISOK Knee Flexion (240°/s) ↔︎
Li 2024 [57]	13	Untrained, Older Adults	HIIT	5 × (3 min at 85% HR max, 3 min at 55% HR max)	Walking	8	Hand Grip Strength ↓
Monsalves-Álvarez 2023 [64]	11	TS Unknown, Overweight/Obese Adults	HIIT	10 × (1 min at 85–90% HR max, 1 min at 50 W)	Cycling	12	Hand Grip Strength ↔︎, Quadriceps Strength ↔︎
Molinari 2022 [92]	9	Resistance/Endurance Trained, Younger Adults	HIIT	10 × (60 s at 85–95% HR max, 3 min walk)	Running	8	Leg Press 1-RM ↑, Knee Extension 1-RM ↔︎
Robinson 2017 [68]	14	Untrained, Younger Adults	HIIT (Young)	4 × (4 min at >90% of VO_2_max, 3 min at 0 W)	Cycling	12	Leg Press 1-RM ↔︎
Robinson 2017 [68]	9	Untrained, Older Adults	HIIT (Old)	4 × (4 min at >90% of VO_2_max, 3 min at 0 W)	Cycling	12	Leg Press 1-RM ↔︎
Schjerve 2008 [93]	14	TS Unknown, Obese, Adults	HIIT	4 × (4 min at HRmax, 3 min at 50–60% of HR max)	Running	12	Leg Press 1-RM ↔︎
Sheykhlouvand 2022 [94]	8	Endurance Trained, Younger Adults	HIIT	6 × (unknown duration at 100% VO_2_peak velocity, 1:1 work to rest ratio)	Kayaking	8	1-RM One Arm Cable Row ↔︎
Sökmen 2018 [95]	20	Recreational, Younger Adults	SIT	40 min (200 m sprint, 200 m walk)	Running	10	ISOK Knee Extension (60°/s) ↔︎, ISOK Knee Flexion (60°/s) ↔︎, ISOK Knee Extension (300°/s) ↑, ISOK Knee Flexion (300°/s) ↑
Song 2023 [96]	10	Trained, Younger Adults	SIT	3 × (7–10 × 15 s at max effort, 15 s rest)	Running	6	Leg Press 1-RM ↔︎
Theofilidis 2021 [73]	7	Recreational, Adults	SIT(Up)	10 × (30 s at 90% MAS +10% grade, 60 s rest)	Running	8	ISOM Knee Extension (65°) ↔︎, ISOM Knee Flexion (30°) ↔︎, ISOK Knee Extension (60°/s) ↔︎
Theofilidis 2021 [73]	7	Recreational, Adults	SIT (Down)	10 × (30 s at 90% MAS −10% grade, 60 s rest)	Running	8	ISOM Knee Extension (65°) ↔︎, ISOM Knee Flexion (30°) ↔︎, ISOK Knee Extension (60°/s) ↔︎
Venegas-Carro 2023 [97]	15	Recreational, Younger Adults	SIT	4–8 × (20–30 s max effort, 10–40 s rest)	Running	6	ISOM Knee Extension (90°) ↔︎, ISOM PF ↔︎
Wong 2024 [98]	11	Recreational, Younger Adults	SIT	1 × (30 s at max effort)	Cycling	6	ISOK Knee Extension (30°/s) ↔︎, ISOK Knee Flexion (30°/s) ↔︎, ISOK Knee Extension (300°/s) ↔︎, ISOK Knee Flexion (300°/s) ↔︎,
Zhang 2024 [99]	14	Sedentary, Younger Adults	HIIT	2–3 × (12–15 × 30 s at max effort, 10 s rest)	Cycling	8	Grip Strength ↔︎

**Table 4:** An overview of the main characteristics of included studies measuring muscle endurance. SIT (sprint interval training), HIIT (high-intensity interval training), S (seconds), Min (minutes), RPE (rating of perceived exertion), VO_2_max/peak (maximal/peak oxygen consumption), MAS (maximum aerobic speed), ISOK (isokinetic), 1-RM (1 repetition maximum), MVC (maximal volitional contraction)), ↑ indicates a significant increase from baseline, ↔︎ indicates no change from baseline.

Author	Sample Size	Training Status/Age	Group Name	Protocol	Modality	Duration (Weeks)	ME Outcome
Bagley 2016 [77]	15	Recreational, Adults	SIT (Female)	4 × (20 s at 175–200% VO_2_peak, 2 min rest)	Cycling	12	Fatigue index—60 reps at max intensity (Knee Extension ISOK 120°/s) ↑
Bagley 2016 [77]	16	Recreational, Adults	SIT (Male)	4 × (20 s at 175–200% VO_2_peak, 2 min rest)	Cycling	12	Fatigue index—60 reps at max intensity (Knee Extension ISOK 120°/s) ↑
Bornath and Kenno 2022 [86]	15	Recreational, Younger Adults	SIT (Female)	10 × (30 s at max effort, 60 s rest)	Battle Ropes	6	Sit-ups (reps completed) ↑, Push-ups (reps completed) ↑
Bornath and Kenno 2022 [86]	18	Recreational, Younger Adults	SIT (Male)	10 × (30 s at max effort, 60 s rest)	Battle Ropes	6	Sit-ups (reps completed) ↑, Push-ups (reps completed) ↑
Buckley 2015 [87]	15	Recreational, Younger Adults	HIIT	6 × (1 min at RPE 9–10/10, 3 min rest)	Rowing	6	70% pre-training squat 1-RM (reps completed) ↔︎
Cao 2024 [88]	12	Sedentary, Younger Adults	SIT	4 × (4 × 30 s at 100–120% MAS, 30 s rest at 50% MAS)	Running	12	Push-ups (reps completed) ↑
Theofilidis 2021 [73]	7	Recreational, Adults	HIIT (Up)	10 × (30 s at 90% MAS +10% grade, 60 s rest)	Running	8	Work till >50% MVC not maintained (Knee Extension ISOK 60°/s) ↑
Theofilidis 2021 [73]	7	Recreational, Adults	HIIT (Down)	10 × (30 s at 90% MAS −10% grade, 60 s rest)	Running	8	Work till >50% MVC not maintained (Knee Extension ISOK 60°/s) ↑

**Table 5:** Pooled weighted effect sizes and percentage gains in FFM, LLM, and quadriceps CSA. FFM (fat-free mass), LLM (leg lean mass), CSA (cross-sectional area), ES (effect size), CI (confidence interval).

Outcome	Weighted ES	95% CI	Weighted %∆
FFM (N = 463)	0.06	−0.03, 0.15	1.17 ± 1.64%
LLM (N = 159)	0.04	0.02, 0.07	0.61 ± 2.36%
Quadriceps CSA (N = 71)	0.36	0.34, 0.37	4.72 ± 1.35%

**Table 6:** Pooled weighted effect sizes and percentage gains in leg press 1-RM, ISOK 60, and ISOM 90 strength.1-RM (one repetition maximum), ISOK 60 (isokinetic strength at 60 degrees/s), ISOM 90 (isometric strength at 90 degrees of knee flexion), ES (effect size), CI (confidence interval).

Outcome	Weighted ES	ES 95% CI	Weighted %∆
Leg Press 1-RM (N = 41)	0.16	0.13, 0.19	3.45 ± 2.19%
ISOK 60 (N = 163)	0.01	−0.02, 0.04	0.35 ± 4.88%
ISOM 90 (N = 108)	0.19	0.15, 0.22	4.94 ± 5.82%

## Data Availability

Data generated or analyzed during this study are provided in full within the published article and its Supplementary Materials.

## Supplementary Materials

The following supporting information can be downloaded at https://www.mdpi.com/article/10.3390/sports13090293/s1, Figure S1: PRISMA 2020 checklist; Table S1: Complete search strategy; Table S2: FFM HIIT versus MICT grade assessment; Table S3: FFM HIIT versus CON grade assessment; Table S4: Leg press 1-RM strength HIIT versus RT grade assessment; Figure S2: FFM HIIT versus MICT leave-one-out analysis; Figure S3: FFM HIIT versus MICT Baujat Plot of heterogeneity contribution; Figure S4: FFM HIIT versus CON leave-one-out analysis; Figure S5: FFM HIIT versus CON Baujat Plot of heterogeneity; Figure S6: Leg press 1-RM strength HIIT versus RT leave-one-out analysis; Figure S7: FFM HIIT versus CON Baujat Plot of heterogeneity contribution.
