# Supplementary material for: Does High-Intensity Interval Training Increase Muscle Strength, Muscle Mass, and Muscle Endurance? A Systematic Review and Meta-Analysis
Source: Sports (Basel). 2025 Sep 1;13(9):293. doi: 10.3390/sports13090293 (PMC12473220; doi:10.3390/sports13090293)
Supplement: Supplementary file 1 [file sports-13-00293-s001.zip › sports-3737868-supplementary.pdf]

Supplemental

Section and Topic	Item #	Checklist item	Location where item is reported
**TITLE**			
Title	1	Identify the report as a systematic review.	Pg1
**ABSTRACT**			
Abstract	2	See the PRISMA 2020 for Abstracts checklist.	Pg1
**INTRODUCTION**			
Rationale	3	Describe the rationale for the review in the context of existing knowledge.	Pg 2-3
Objectives	4	Provide an explicit statement of the objective(s) or question(s) the review addresses.	Pg 3
**METHODS**			
Eligibility criteria	5	Specify the inclusion and exclusion criteria for the review and how studies were grouped for the syntheses.	Pg 3 (2.2)
Information sources	6	Specify all databases, registers, websites, organisations, reference lists and other sources searched or consulted to identify studies. Specify the date when each source was last searched or consulted.	Pg 3 (2.3)
Search strategy	7	Present the full search strategies for all databases, registers and websites, including any filters and limits used.	Pg 3 (2.3)
Selection process	8	Specify the methods used to decide whether a study met the inclusion criteria of the review, including how many reviewers screened each record and each report retrieved, whether they worked independently, and if applicable, details of automation tools used in the process.	Pg 3-4 (2.4)
Data collection process	9	Specify the methods used to collect data from reports, including how many reviewers collected data from each report, whether they worked independently, any processes for obtaining or confirming data from study investigators, and if applicable, details of automation tools used in the process.	Pg 4 (2.5)
Data items	10a	List and define all outcomes for which data were sought. Specify whether all results that were compatible with each outcome domain in each study were sought (e.g. for all measures, time points, analyses), and if not, the methods used to decide which results to collect.	Pg 4 (2.5)
	10b	List and define all other variables for which data were sought (e.g. participant and intervention characteristics, funding sources). Describe any assumptions made about any missing or unclear information.	Pg 4 (2.5)
Study risk of bias assessment	11	Specify the methods used to assess risk of bias in the included studies, including details of the tool(s) used, how many reviewers assessed each study and whether they worked independently, and if applicable, details of automation tools used in the process.	Pg 4 (2.6)
Effect measures	12	Specify for each outcome the effect measure(s) (e.g. risk ratio, mean difference) used in the synthesis or presentation of results.	Pg 4-5 (2.8)
Synthesis methods	13a	Describe the processes used to decide which studies were eligible for each synthesis (e.g. tabulating the study intervention characteristics and comparing against the planned groups for each synthesis (item #5)).	Pg 4-5 (2.8)
	13b	Describe any methods required to prepare the data for presentation or synthesis, such as handling of missing summary statistics, or data conversions.	Pg 4-5 (2.8)
	13c	Describe any methods used to tabulate or visually display results of individual studies and syntheses.	Pg 4-5 (2.8)
	13d	Describe any methods used to synthesize results and provide a rationale for the choice(s). If meta-analysis was performed, describe the model(s), method(s) to identify the presence and extent of statistical heterogeneity, and software package(s) used.	Pg 4-5 (2.8)

Section and Topic	Item #	Checklist item	Location where item is reported
	13e	Describe any methods used to explore possible causes of heterogeneity among study results (e.g. subgroup analysis, meta-regression).	Pg 4-5 (2.8)
	13f	Describe any sensitivity analyses conducted to assess robustness of the synthesized results.	Pg 4-5 (2.8)
Reporting bias assessment	14	Describe any methods used to assess risk of bias due to missing results in a synthesis (arising from reporting biases).	Pg 4 (2.5)
Certainty assessment	15	Describe any methods used to assess certainty (or confidence) in the body of evidence for an outcome.	Pg 4 (2.7)
**RESULTS**			
Study selection	16a	Describe the results of the search and selection process, from the number of records identified in the search to the number of studies included in the review, ideally using a flow diagram.	Pg 6 – (Figure 1)
	16b	Cite studies that might appear to meet the inclusion criteria, but which were excluded, and explain why they were excluded.	Pg (3.2)
Study characteristics	17	Cite each included study and present its characteristics.	Pg 6-14 (Table 1-3)
Risk of bias in studies	18	Present assessments of risk of bias for each included study.	Pg 15 – Figure 2
Results of individual studies	19	For all outcomes, present, for each study: (a) summary statistics for each group (where appropriate) and (b) an effect estimate and its precision (e.g. confidence/credible interval), ideally using structured tables or plots.	Pg 7-14 (Table 1-3)
Results of syntheses	20a	For each synthesis, briefly summarise the characteristics and risk of bias among contributing studies.	Pg 16-17 (3.4.1, 3.4.2, 3.4.3, Figure 3-5)
	20b	Present results of all statistical syntheses conducted. If meta-analysis was done, present for each the summary estimate and its precision (e.g. confidence/credible interval) and measures of statistical heterogeneity. If comparing groups, describe the direction of the effect.	Pg 4-5 (2.8)
	20c	Present results of all investigations of possible causes of heterogeneity among study results.	Figure S3, S5, S7
	20d	Present results of all sensitivity analyses conducted to assess the robustness of the synthesized results.	Figure S2, S4, S6
Reporting biases	21	Present assessments of risk of bias due to missing results (arising from reporting biases) for each synthesis assessed.	Pg 4 (2.5)
Certainty of evidence	22	Present assessments of certainty (or confidence) in the body of evidence for each outcome assessed.	Table S3-S5
**DISCUSSION**			
Discussion	23a	Provide a general interpretation of the results in the context of other evidence.	Pg 18 (4)
	23b	Discuss any limitations of the evidence included in the review.	Pg 20 (4.5)
	23c	Discuss any limitations of the review processes used.	Pg 20 (4.5)
	23d	Discuss implications of the results for practice, policy, and future research.	Pg 20-21 (4.6)
**OTHER INFORMATION**			
Registration and protocol	24a	Provide registration information for the review, including register name and registration number, or state that the review was not registered.	Pg 21
	24b	Indicate where the review protocol can be accessed, or state that a protocol was not prepared.	Pg 21
	24c	Describe and explain any amendments to information provided at registration or in	Pg 5 (2.9)

Section and Topic	Item #	Checklist item	Location where item is reported
		the protocol.	
Support	25	Describe sources of financial or non-financial support for the review, and the role of the funders or sponsors in the review.	Pg 21
Competing interests	26	Declare any competing interests of review authors.	Pg 21
Availability of data, code and	27	Report which of the following are publicly available and where they can be found: template data collection forms; data extracted from included studies; data used for all analyses; analytic code; any other materials used in the review.	Pg 21
other materials			Supplementary materials

Figure S1 – PRISMA Checklist. Pg (page)

	EMBASE	OVID Medline	SPORTDiscus	CINAHL
**1**	High-intensity interval training.sh. or (High-intensity aerobic interval training or Interval exercise or High-intensity intermittent training or Interval training or Sprint-interval training or High-intensity intermittent exercise or HIIT or SIT or High-intensity interval training or Aerobic interval training).mp.	High-intensity interval training.sh. or (SIT or HIIT or High-intensity aerobic interval training or Interval exercise or High-intensity intermittent training or High-intensity interval training or Interval training or Sprint-interval training or High-intensity intermittent exercise or aerobic interval training).mp.	SU(High-intesnity interval training or Interval training) or KW ((High-intesnity interval training or Interval training or Sprint interval training or High-intensity intermittent exercise or Aerobic interval training or SIT or HIIT)	SU(High-intesnity interval training) or (High-intesnity interval training or Interval training or Sprint interval training or High-intensity intermittent exercise or Aerobic interval training or SIT or HIIT))
**2**	Muscle strength.sh. or (Strength or Musc* strength).mp.	Muscle strength.sh. or (Musc* strength or Strength).mp.	SU(Muscle strength) or KW ((Musc*strength or Strength))	SU(Muscle strength) or ((Musc*strength or Strength))
**3**	(Muscle hypertrophy or Muscle Growth or Hypertrophy).sh. or (Musc*hypertrophy or Musc*Growth or Hypertrophy).mp.	(Muscle hypertrophy or Hypertrophy or Muscle Growth).sh. or (Musc* hypertrophy or Musc* Growth or Hypertrophy).mp.	SU(Muscle hypertrophy or Hypertrophy or Muscle growth))or KW ((Musc*hypertrophy or Hypertrophy or Musc* growth))	SU(Muscle hypertrophy or Hypertrophy)) or ((Musc*hypertrophy or Hypertrophy or Musc* growth))
**4**	(Musc*endurance or Strength endurance or Musc*fatiguability or Fatigue resistance).mp.	(Musc*endurance or Strength endurance or Musc*fatiguability or fatigue resisitance).mp.	KW ((Musc*endurance or Strength endurance or Musc* fatiguability or Fatigue resistance))	(Musc*endurance or Strength endurance or Musc* fatiguability or Fatigue resistance)
**5**	1 and (2 or 3 or 4)	1 and (2 or 3 or 4)	S1 and (S2 or S3 or S4)	S1 and (S2 or S3 or S4)

Table S1 – Complete Search Strategy. HIIT (High-intensity interval training), SIT (Sprint interval training), SU (Subject), KW (keyword), Musc* (Muscle)

GRADE criteria	Rating	Footnotes (explain reasons for down- or upgrading)	Quality of the evidence (Circle one)
Outcome: FFM			
Study design		All RCTs starts at high	⊕ Low
Risk of Bias *(use the Cochrane Risk of Bias tables and figures)*	serious (-1)	Moderate-high ROB for all studies	
Inconsistency	No	Large overlap in confidence intervals	
Indirectness	No	All studies address the research question directly, I^2=0%	
Imprecision	serious (-1)	Confidence intervals cross zero	
Publication Bias	Undetected		
Other (upgrading factors, circle all that apply)	Large effect (+1 or +2)<br>Dose response (+1 or +2)<br>No Plausible confounding (+1 or +2)		

Table S2 – FFM HIIT Versus MICT Grade Assessment. RCT (randomized control trial), ROB (risk of bias), FFM (Fat free mass). [1-10].

GRADE criteria	Rating	Footnotes (explain reasons for down- or upgrading)	Quality of the evidence (Circle one)
Outcome: FFM			
Study design		All RCTs starts at high	⊕ Low
Risk of Bias *(use the Cochrane Risk of Bias tables and figures)*	serious (-1)	Moderate-high ROB for all studies	
Inconsistency	No	Large overlap in confidence intervals	
Indirectness	No	All studies address the research question directly, I^2=0%	
Imprecision	serious (-1)	Confidence intervals cross zero	
Publication Bias	Undetected		
Other (upgrading factors, circle all that apply)	Large effect (+1 or +2)<br>Dose response (+1 or +2)<br>No Plausible confounding (+1 or +2)		

Table S3– FFM HIIT Versus CON Grade Assessment. FFM HIIT Versus MICT Grade Assessment. RCT (randomized control trial), ROB (risk of bias), FFM (Fat free mass). [2, 4, 11-13].

GRADE criteria	Rating	Footnotes<br>(explain reasons for down- or upgrading)	Quality of the evidence<br>(Circle one)
Outcome: 1-RM Leg Press Strength			
Study design		All RCTs starts at high	⊕<br>Low
Risk of Bias<br>*(use the Cochrane Risk of Bias tables and figures)*	serious (-1)	Moderate-high ROB for all studies	
Inconsistency	No	Large overlap in confidence intervals	
Indirectness	No	All studies address the research question directly, I^2=0%	
Imprecision	serious (-1)	Confidence intervals cross zero	
Publication Bias	Undetected		
Other<br>(upgrading factors, circle all that apply)	Large effect (+1 or +2)<br>Dose response (+1 or +2)<br>No Plausible confounding (+1 or +2)		

Table S4– Leg Press 1-RM Strength HIIT Versus RT Grade Assessment. FFM HIIT Versus MICT Grade Assessment. RCT (randomized control trial), ROB (risk of bias), FFM (Fat free mass). [14-16].

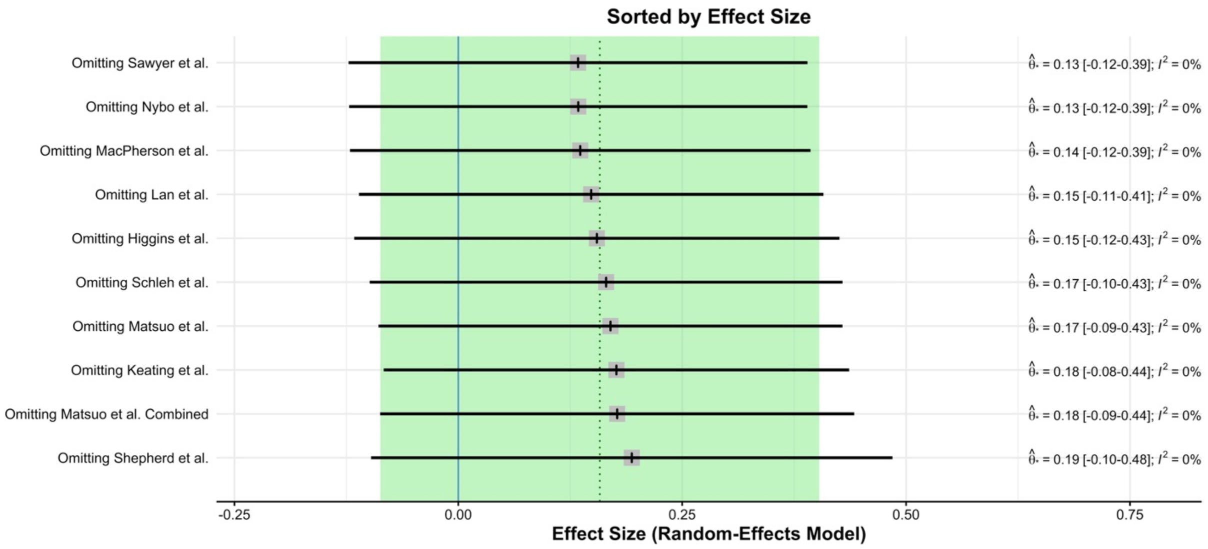

Figure S2 – FFM HIIT Versus MICT Leave-One-Out Analysis [1-10].

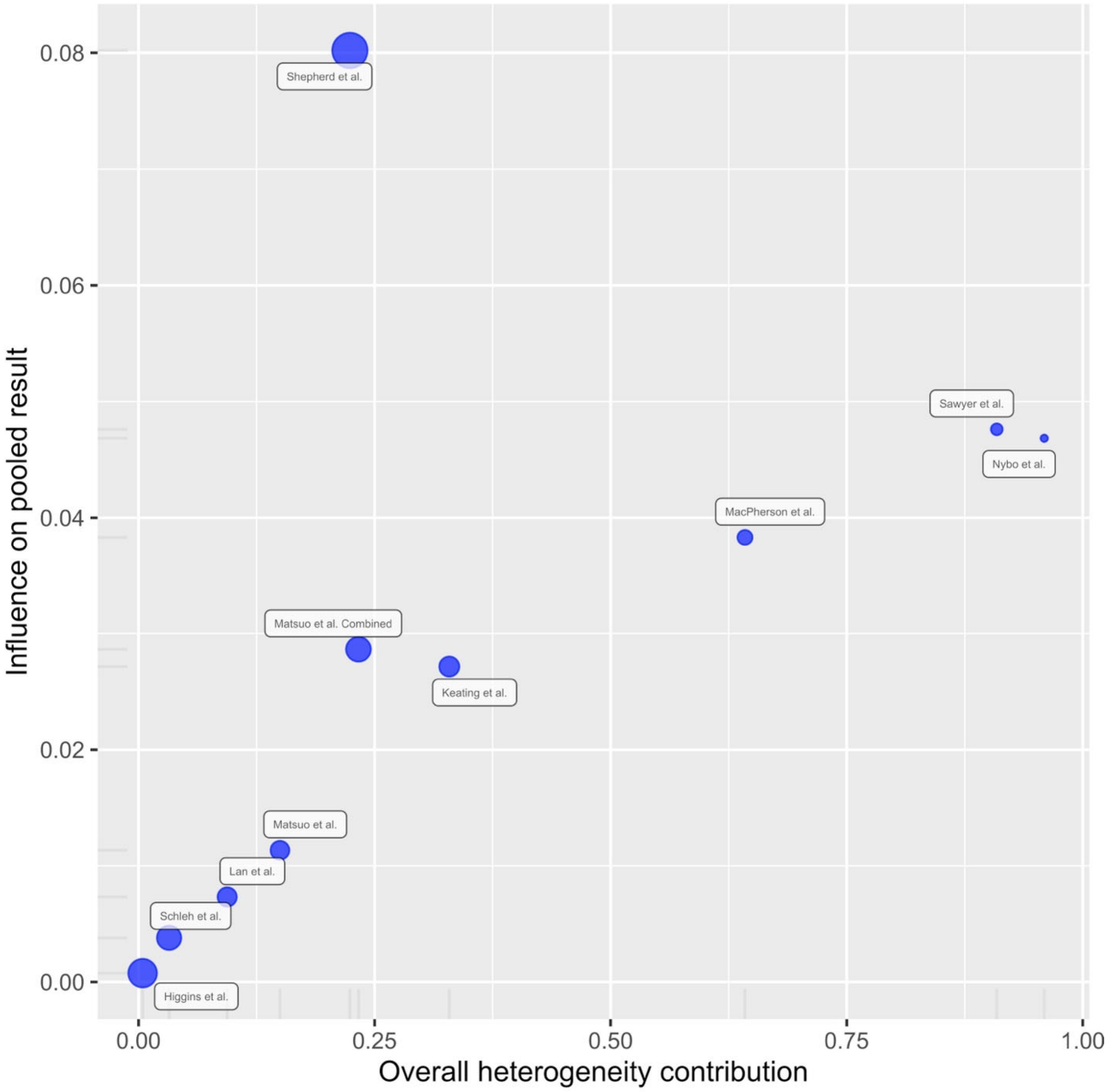

Figure S3 - FFM HIIT Versus MICT Baujat Plot of Heterogeneity Contribution [1-10].

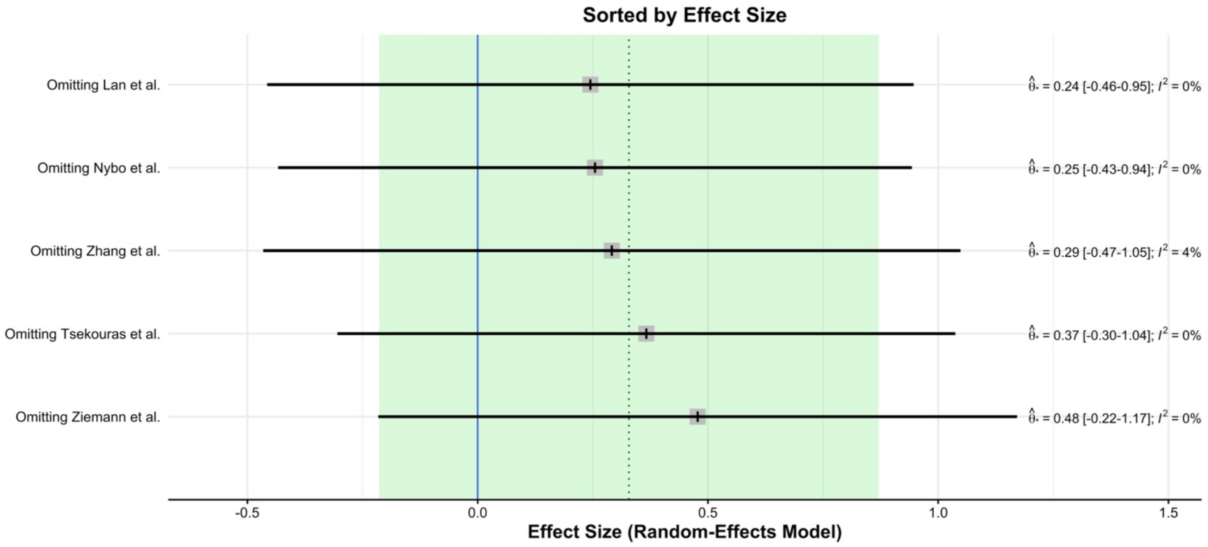

Figure S4 – FFM HIIT Versus CON Leave-One-Out Analysis [2, 4, 11-13]

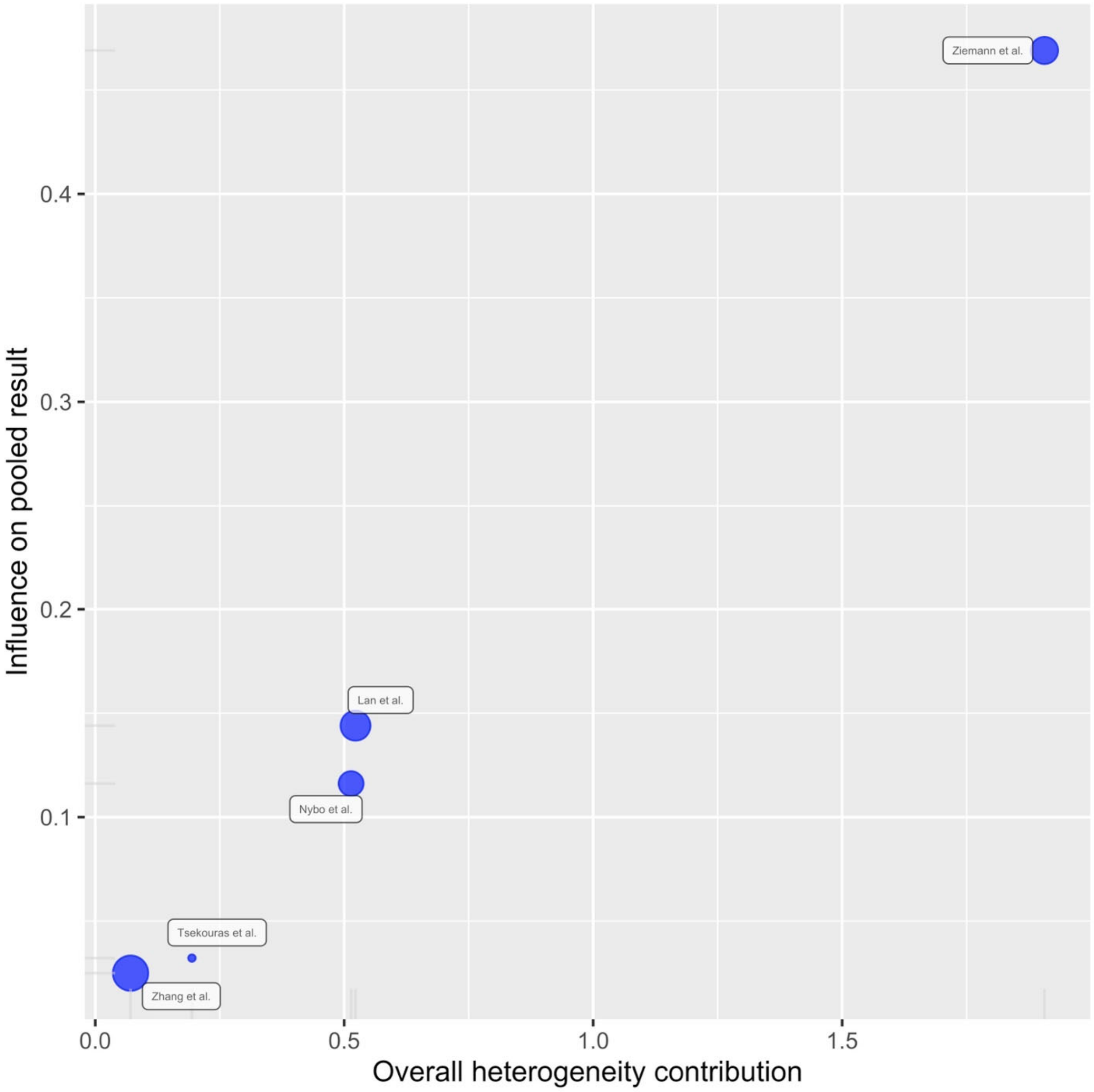

Figure S5 - FFM HIIT Versus CON Baujat Plot of Heterogeneity Contribution [2, 4, 11-13]

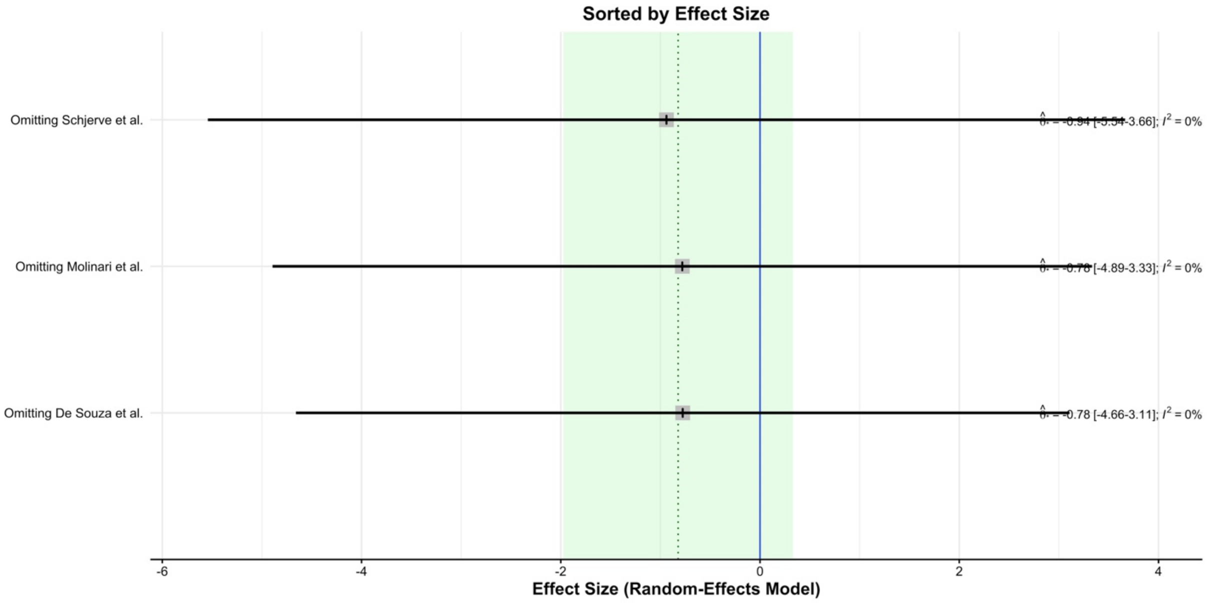

Figure S6 – Leg Press 1-RM Strength HIIT Versus RT Leave-One-Out Analysis [14-16]

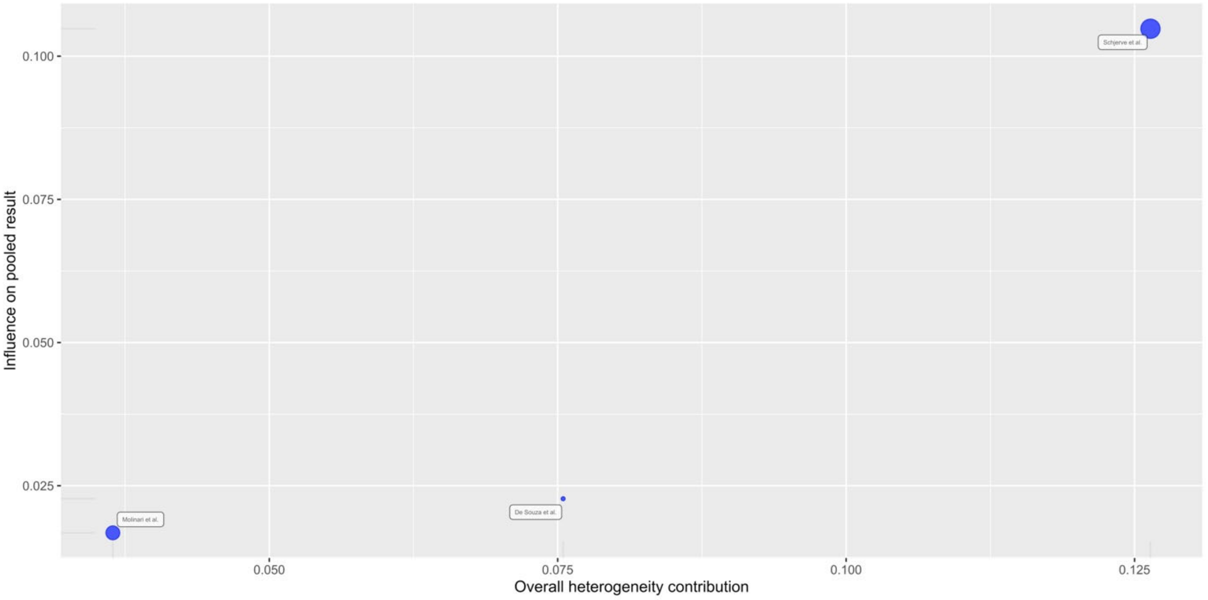

Figure S7 - FFM HIIT Versus CON Baujat Plot of Heterogeneity Contribution [14-16].

References

[1] B. J. Sawyer, W. J. Tucker, D. M. Bhammar, J. R. Ryder, K. L. Sweazea, and G. A. Gaesser, "Effects of high-intensity interval training and moderate-intensity continuous training on endothelial function and cardiometabolic risk markers in obese adults," *J Appl Physiol*, vol. 121, no. 1, pp. 279–288, Jul. 2016, doi: 10.1152/japplphysiol.00024.2016.

[2] L. Nybo *et al.*, "High-Intensity Training versus Traditional Exercise Interventions for Promoting Health," *Medicine & Science in Sports & Exercise*, vol. 42, no. 10, pp. 1951–1958, Oct. 2010, doi: 10.1249/MSS.0b013e3181d99203.

[3] R. E. K. Macpherson, T. J. Hazell, T. D. Olver, D. H. Paterson, and P. W. R. Lemon, "Run Sprint Interval Training Improves Aerobic Performance but Not Maximal Cardiac Output," *Medicine & Science in Sports & Exercise*, vol. 43, no. 1, pp. 115–122, Jan. 2011, doi: 10.1249/MSS.0b013e3181e5eacd.

[4] C. Lan, Y. Liu, and Y. Wang, "Effects of different exercise programs on cardiorespiratory fitness and body composition in college students," *Journal of Exercise Science & Fitness*, vol. 20, no. 1, pp. 62–69, Jan. 2022, doi: 10.1016/j.jesf.2021.12.004.

[5] M. F. Higgins, R. S. James, and M. J. Price, "The effects of sodium bicarbonate ($NaHCO_3$) ingestion on high intensity cycling capacity," *Journal of Sports Sciences*, vol. 31, no. 9, pp. 972–981, May 2013, doi: 10.1080/02640414.2012.758868.

[6] M. W. Schleh *et al.*, "Both moderate- and high-intensity exercise training increase intramyocellular lipid droplet abundance and modify myocellular distribution in adults with obesity," *American Journal of Physiology-Endocrinology and Metabolism*, vol. 325, no. 5, pp. E466–E479, Nov. 2023, doi: 10.1152/ajpendo.00093.2023.

[7] T. Matsuo *et al.*, "Low-volume, high-intensity, aerobic interval exercise for sedentary adults: $$\dot{V}$$ V ˙ O2max, cardiac mass, and heart rate recovery," *Eur J Appl Physiol*, vol. 114, no. 9, pp. 1963–1972, Sep. 2014, doi: 10.1007/s00421-014-2917-7.

[8] T. Matsuo *et al.*, "Effects of a Low-Volume Aerobic-Type Interval Exercise on V˙O2max and Cardiac Mass," *Medicine & Science in Sports & Exercise*, vol. 46, no. 1, p. 42, Jan. 2014, doi: 10.1249/MSS.0b013e3182a38da8.

[9] S. E. Keating *et al.*, "Continuous Exercise but Not High Intensity Interval Training Improves Fat Distribution in Overweight Adults," *Journal of Obesity*, vol. 2014, pp. 1–12, 2014, doi: 10.1155/2014/834865.

[10] S. O. Shepherd *et al.*, "Low-Volume High-Intensity Interval Training in a Gym Setting Improves Cardio-Metabolic and Psychological Health," *PLoS ONE*, vol. 10, no. 9, p. e0139056, Sep. 2015, doi: 10.1371/journal.pone.0139056.

[11] B. Zhang *et al.*, "The effect of different high-intensity interval training protocols on cardiometabolic and inflammatory markers in sedentary young women: A randomized controlled trial," *Journal of Sports Sciences*, vol. 42, no. 8, pp. 751–762, Apr. 2024, doi: 10.1080/02640414.2024.2363708.

[12] Y. E. Tsekouras, F. Magkos, Y. Kellas, K. N. Basioukas, S. A. Kavouras, and L. S. Sidossis, "High-intensity interval aerobic training reduces hepatic very low-density lipoprotein-triglyceride secretion rate in men," *American Journal of Physiology-Endocrinology and Metabolism*, vol. 295, no. 4, pp. E851–E858, Oct. 2008, doi: 10.1152/ajpendo.90545.2008.

[13] E. Ziemann, T. Grzywacz, M. Łuszczyk, R. Laskowski, R. A. Olek, and A. L. Gibson, "Aerobic and Anaerobic Changes with High-Intensity Interval Training in Active College-Aged Men," *Journal of Strength and Conditioning Research*, vol. 25, no. 4, pp. 1104–1112, Apr. 2011, doi: 10.1519/JSC.0b013e3181d09ec9.

[14] I. E. Schjerve *et al.*, "Both aerobic endurance and strength training programmes improve cardiovascular health in obese adults," *Clinical Science*, vol. 115, no. 9, pp. 283–293, Nov. 2008, doi: 10.1042/CS20070332.

[15] T. Molinari, T. Molinari, R. Rabello, and R. Rodrigues, "Effects of 8 weeks of high-intensity interval training or resistance training on muscle strength, muscle power and cardiorespiratory responses in trained young men," *Sport Sci Health*, vol. 18, no. 3, pp. 887–896, Sep. 2022, doi: 10.1007/s11332-021-00872-2.
[16] E. de Souza *et al.*, "Molecular Adaptations to Concurrent Training," *Int J Sports Med*, vol. 34, no. 03, pp. 207–213, Oct. 2012, doi: 10.1055/s-0032-1312627.

Disclaimer/Publisher's Note: The statements, opinions and data contained in all publications are solely those of the individual author(s) and contributor(s) and not of MDPI and/or the editor(s). MDPI and/or the editor(s) disclaim responsibility for any injury to people or property resulting from any ideas, methods, instructions or products referred to in the content.

References
